# Truncating *RELA* variants drive autoinflammation and autoimmunity by impairing the negative feedback control of NF-**κ**B

**DOI:** 10.1172/jci.insight.200002

**Published:** 2026-08-11

**Authors:** Nadja Lucas, Sophia Weidler, Antonia A. Eicher, Baerbel Keller, Özlem Satirer, Adam Desrochers, Timothy J.S. Ramnarine, Mohammad Mokhtari, Sophie Elstner, Timmy Strauss, Simon W. Mages, Arek Kendirli, Oana Cristina Buzoianu, Tobias B. Haack, Lina Igel, Tim Niehues, Sandra von Hardenberg, Maria Fasshauer, Rami Abou Jamra, Hagen Ott, Ulrike Hüffmeier, Catharina Schuetz, Marisa Bijwaard, Susan Wagner, Paulina Switala, Sarah Koss, Jurek Schultz, Stefanie Kretschmer, Jasmin Kümmerle-Deschner, Christine Wolf, Johanna Klughammer, Min Ae Lee-Kirsch

**Affiliations:** 1 Department of Pediatrics, Medizinische Fakultät Carl Gustav Carus, Technische Universität Dresden, Dresden, Germany.; 2 Gene Center and Department of Biochemistry, Ludwig-Maximilians-Universität München, Munich, Germany.; 3 Department of Rheumatology and Clinical Immunology, and; 4 Center for Chronic Immunodeficiency (CCI), Medical Center - University of Freiburg, Faculty of Medicine, University of Freiburg, Freiburg, Germany.; 5 Division of Paediatric Rheumatology and Autoinflammation Reference Centre Tübingen, Department of Paediatrics, University Hospital Tübingen, Tübingen, Germany.; 6 Institute of Clinical Neuroimmunology, University Hospital, Ludwig-Maximilians-Universität München, Munich, Germany.; 7 Institute of Medical Genetics and Applied Genomics, University Hospital Tübingen, Tübingen, Germany.; 8 Department of Pediatrics, Helios Klinik Krefeld, Krefeld, Germany.; 9 Department of Human Genetics, Hannover Medical School, Hannover, Germany.; 10 Hospital for Children and Adolescents, Hospital St. Georg, Academic Teaching Hospital of the University of Leipzig, Leipzig, Germany.; 11 Institute of Human Genetics, University of Leipzig Medical Center, Leipzig, Germany.; 12 Section of Pediatric Dermatology, Munich University Center for Children with Medical and Developmental Complexity, München, Germany.; 13 Institute of Human Genetics, Universitätsklinikum Erlangen, Friedrich-Alexander-Universität Erlangen-Nürnberg, Erlangen, Germany.; 14 German Center for Child and Adolescent Health (DZKJ), partner site Leipzig/Dresden, Dresden, Germany.; 15 Department of Pediatric Surgery, Medizinische Fakultät Carl Gustav Carus, and; 16 University Center for Rare Diseases, Medizinische Fakultät Carl Gustav Carus, Technische Universität Dresden, Dresden, Germany.

**Keywords:** Immunology, Inflammation, Innate immunity, Molecular genetics, Rheumatology

## Abstract

The NF-κB signaling pathway coordinates inflammation, cell survival, and proliferation, while restraining excessive cell death to maintain immune homeostasis. Truncating mutations in *RELA*, encoding the NF-κB subunit p65, have been linked to autoinflammation and autoimmunity, but the underlying mechanisms remain incompletely defined. We investigated 6 patients from 5 unrelated families carrying heterozygous truncating *RELA* variants. Despite reduced p65 expression, patients exhibited a broad spectrum of inflammatory manifestations alongside elevated baseline and stimulus-induced proinflammatory cytokines. Functional analyses in patient-derived cells and mutant *RELA*-KI models showed that upstream NF-κB signaling was intact, but induction of inhibitory regulators such as IκBα and A20 was impaired. This defective feedback control shifted immune homeostasis toward amplified inflammatory responses that depended on the residual activity of the remaining functional *RELA* allele. Single-cell transcriptomics revealed distinct cell type–specific consequences: monocytes displayed constitutive type I IFN and NF-κB activation, B cells retained partial compensatory signaling, and T and NK cells exhibited transcriptional signatures of cell death pathways. Patient fibroblasts and mutant *RELA*-KI cells further confirmed enhanced TNF-induced inflammatory gene expression and hypersensitivity to apoptosis and necroptosis. These findings establish *RELA* haploinsufficiency as a cause of systemic immune dysregulation and link defective NF-κB feedback control to unchecked inflammation and inflammatory cell death.

## Abstract 2

Inherited RELA mutations disable a key brake on inflammation, causing excessive immune activation and inflammatory cell death that drive autoinflammatory and autoimmune disease.

## Graphical abstract

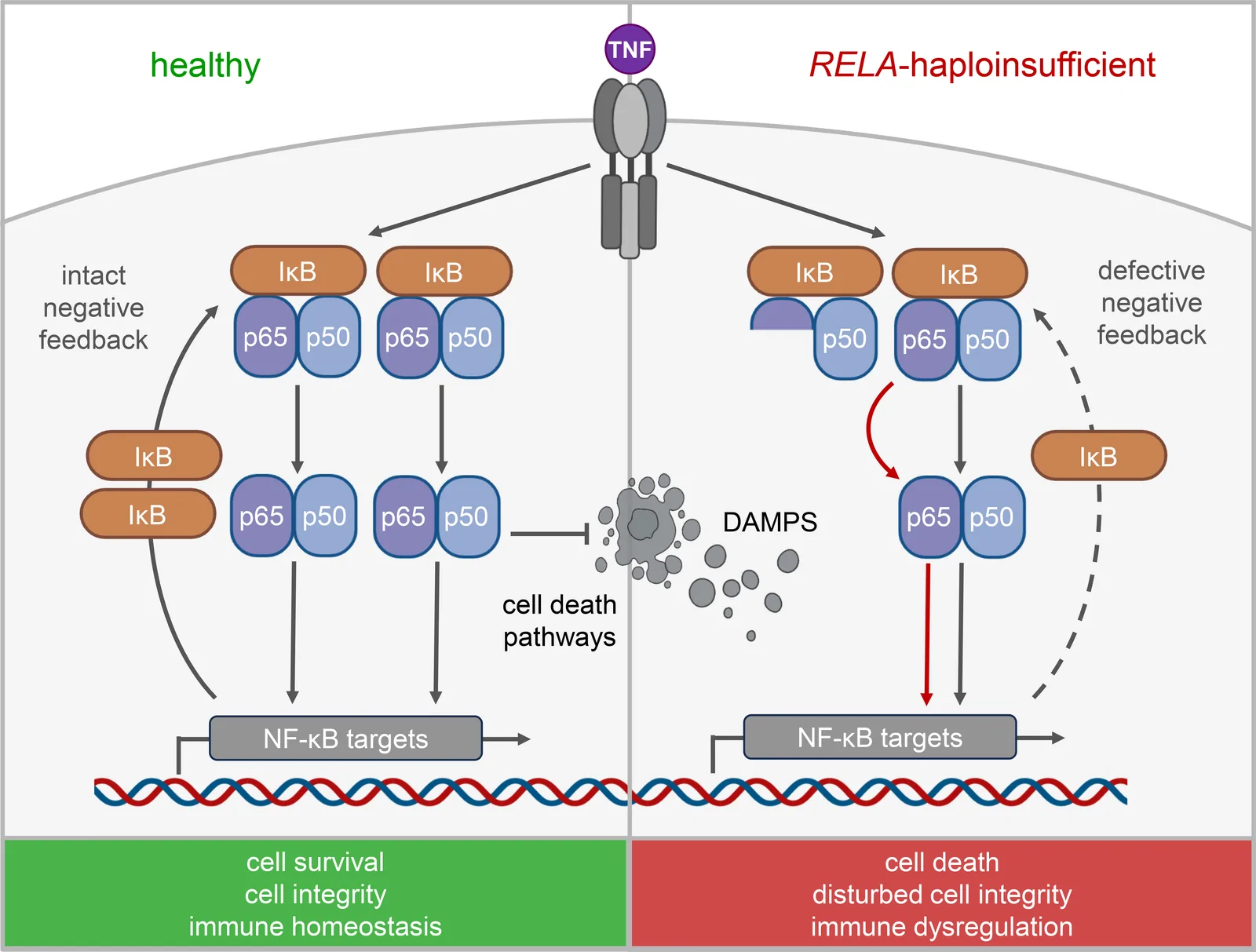

## Introduction

The NF-κB signaling pathway is a complex regulatory network that plays a central role in immune responses, inflammation, and cell survival (1–3). It functions primarily as a transcriptional activator of genes involved in both innate and adaptive immunity and is critical for maintaining immune homeostasis and responding to cellular stress. The NF-κB family consists of 5 structurally related proteins: RelA (p65), RelB, c-Rel, NF-κB1 (p105/p50), and NF-κB2 (p100/p52) (1). All subunits share a conserved Rel homology domain responsible for dimerization, DNA binding, and interaction with inhibitory IκB proteins. Among these, RelA, RelB, and c-Rel possess C-terminal transactivation domains (TADs) and act as transcriptional activators (1). In contrast, p50 and p52 lack TADs and must form heterodimers with TAD-containing subunits to activate gene transcription. In the absence of stimulation, NF-κB dimers are sequestered in the cytoplasm through interactions with IκBs, such as IκBα, IκBβ, and IκBε, which mask their nuclear localization sequences, thereby preventing nuclear translocation (1, 2). The canonical NF-κB pathway is activated by a wide range of stimuli, including TNF, IL-1, TLR ligands, and antigens (1, 2).

Upon activation of these receptors, upstream kinases converge on the IκB kinase (IKK) complex, composed of IKKα, IKKβ, and the regulatory subunit NEMO (IKKγ). The activated IKK complex phosphorylates IκB proteins, marking them for ubiquitination and proteasomal degradation (1, 2). This process releases the NF-κB dimers, most commonly p65/p50, which translocate into the nucleus, bind κB enhancer elements in target gene promoters, and induce transcription of a broad array of genes involved in inflammation, cell proliferation, survival, and immune regulation (1, 2).

The noncanonical NF-κB pathway, activated by CD40, BAFFR, or LTβR, relies on NIK-driven processing of p100 to p52, which dimerizes with RelB to form the active complex (3). Both pathways are tightly regulated by negative feedback mechanisms. In the canonical pathway, this includes NF-κB–induced expression of IκBα and deubiquitinases such as *TNFAIP3* (A20), CYLD, and OTULIN, which act to terminate upstream signaling cascades (4). Dysregulation of NF-κB signaling is associated with a range of pathologies, including chronic inflammation, autoimmune diseases, and cancer (1, 2).

Truncating *RELA* variants have been implicated in a spectrum of autoinflammatory and autoimmune disorders, including Behçet-like mucocutaneous ulcerations (5–9), inflammatory bowel disease (10, 11), juvenile idiopathic arthritis (11), autoimmune hematological conditions (12), and early-onset systemic lupus erythematosus (SLE) (13). Although both haploinsufficiency, leading to increased sensitivity to TNF, and dominant-negative effects, associated with heightened TLR7 signaling, have been proposed as pathogenic mechanisms (5, 11, 12), the precise molecular basis of *RELA*-associated immune dysregulation remains incompletely understood.

We investigated 6 patients from 5 unrelated families presenting with overlapping autoinflammatory and autoimmune features, all harboring truncating *RELA* mutations. Our findings demonstrate that these mutations lead to a loss of NF-κB signaling function, disrupting the finely tuned balance between NF-κB–mediated pro-survival/proinflammatory and cell death pathways. This imbalance results in a shift toward apoptosis and necroptosis, ultimately driving hyperinflammatory responses.

## Results

### Autoinflammation and autoimmunity in patients with truncating RELA mutations.

**Figure 1:**
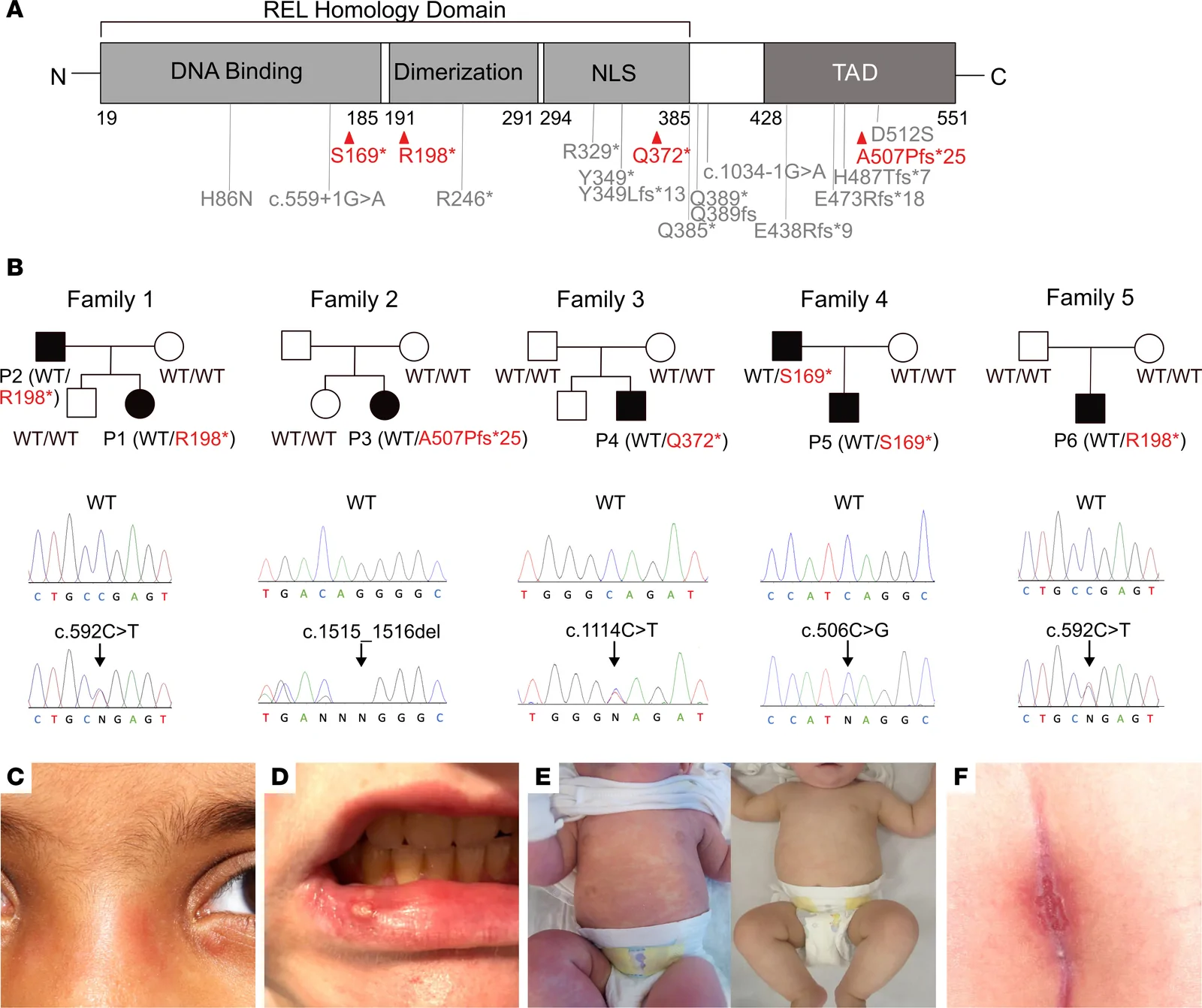
Genetic and clinical findings in patients with truncating *RELA* variants. (**A**) Schematic p65 domain architecture. NLS, nuclear localization signal; TAD, transactivation domain. Newly identified (red) and previously described (gray) pathogenic *RELA* variants. (**B**) Pedigrees and sequence pherograms depicting *RELA* variants. (**C**) Erythema nodosum in periorbital region of P1. (**D**) Oral aphthous ulcer in P3. (**E**) Generalized refractory eczema in P5 and marked clinical improvement under systemic dupilumab. There was no clinical data available for the father of P5. (**F**) Perianal ulcer in P6.

We studied 6 patients from 5 unrelated families (Figure 1, A and B) who presented with signs of autoinflammation and autoimmunity of varying degrees of severity (Table 1). Patient 1 (P1) presented at the age of 3 years with recurrent fevers; arthralgias; and painful erythematous nodules on the head (Figure 1C), face, and back. Histology of a nodule biopsy was consistent with panniculitis. At the age of 5, she developed lichenoid ulcerating skin lesions in the genital area and osteitis of the head, neck, and mandibular joints. Laboratory tests showed elevated levels of erythrocyte sedimentation rate (ESR), C-reactive protein (CRP), IL-6, soluble IL-2 receptor (sIL-2R), serum amyloid a (SAA), and calprotectin. P1 had hypergammaglobulinemia, elevated antinuclear antibodies (ANA; 1:160 to 1:1,100) and an elevated IFN score. Immunophenotyping showed an expansion of IgM^++^CD38^++^ transitional B cells and normal T cells. Genetic testing revealed a heterozygous nonsense variant in the *RELA* gene (NM_021975.4; c.592C>T, p.Arg198*) (Figure 1A). The variant was also identified in the patient’s father (P2), who developed autoimmunity at the age of 26 years with vitiligo on the legs, trunk, and face. Like his daughter, he also had an elevated IFN signature. P3 presented with inflammatory symptoms at the age of 15 years, with recurrent oral (Figure 1D) and genital ulcers; malar rash; arthralgia of the wrists, interphalangeal joints, knees, and back; and intermittent abdominal pain with diarrhea and elevated calprotectin. A lesional skin biopsy showed inflammatory infiltrates, vasculitis, and microabscesses compatible with Behçet′s disease. Laboratory findings included intermittent lymphopenia with unremarkable lymphocyte differentiation, hypergammaglobulinemia, elevated ANA (1:1,280 to 1:20,480) and SS-B/La antibodies (strongly positive), and an increased IFN score. Genetic testing revealed a heterozygous de novo *RELA* variant (c.1515_1516del; p.Ala507Profs*25) resulting in a frameshift and premature stop codon (Figure 1A). P4 developed recurrent fevers from the age of 18 months associated with fatigue, intermittent abdominal pain and diarrhea, and oral aphthae. Laboratory tests repeatedly showed increased CRP, sIL-2R, SAA, calprotectin, and ANA (1:160). Immunophenotyping showed increased γδ T cells, CD38^++^CD138^+^ plasma cells, and CD19^+^CD38^++^CD138^–^ plasmablasts, indicative of autoimmunity. Exome analysis of P4 revealed a heterozygous de novo *RELA* variant (c.1114C>T, p.Gln372*). P5 presented with severe protein-losing enteropathy at 3 months of age. He also developed refractory atopic eczema from the age of 5 months, which progressed to generalized erythroderma (Figure 1E). Laboratory investigations revealed iron deficiency anemia, hypalbuminemia, hypogammaglobulinemia with normal B cell counts, and IgE-mediated sensitization to several foods. P5 also had hypothyroidism requiring hormone replacement. Treatment with dupilumab (IL-4/IL-13 antibody) led to a marked improvement of the skin (Figure 1E), which still required intensive topical treatment with pimecrolimus and prednicarbate. On a hypoallergenic amino acid–based formula, enteropathy and weight gain improved. Genetic testing identified a heterozygous *RELA* nonsense variant (c.506C>G, p.Ser169*). P6 developed fever episodes and skin rashes in the genital area at 10 months of age (Figure 1F), along with elevated CRP. Whole-exome sequencing revealed a heterozygous de novo *RELA* variant (c.592C>T, p.Arg198*), which had also been detected in the unrelated patients P1 and P2. None of the patients showed an increased susceptibility to infections.

All identified *RELA* variants had not previously been described in the context of an inflammatory disease phenotype and were absent from the gnomAD v4.1.0 database, except for A507Pfs*25 with a very low allele frequency of 0.000006 (Table 1). Given a loss of function intolerance (pLI) score of 1 for the *RELA* gene (https://gnomad.broadinstitute.org/gene/ENSG00000173039?dataset=gnomad_r4), all identified variants were predicted to be deleterious. Consistent with previous reports (13), lymphoblastoid cells from P1–P4 carrying truncating *RELA* mutations showed expression of mutant p65, and WT p65 expression was reduced compared with cells from healthy controls (Supplemental Figure 1A; supplemental material available online with this article; https://doi.org/10.1172/jci.insight.200002DS1). As expected, the p65^R198*^ mutant, which lacks both the Rel homology domain required for dimerization and the transactivation domain, was unable to form homodimers with WT p65 or heterodimers with p50 and could not transactivate an NF-κB reporter gene. Likewise, the truncating mutants S169*, Q372*, and A507Pfs*25 also showed significantly reduced reporter activity (Supplemental Figure 1, B and C).

### Increased levels of proinflammatory cytokines at baseline and in response to immune stimulation.

**Figure 2:**
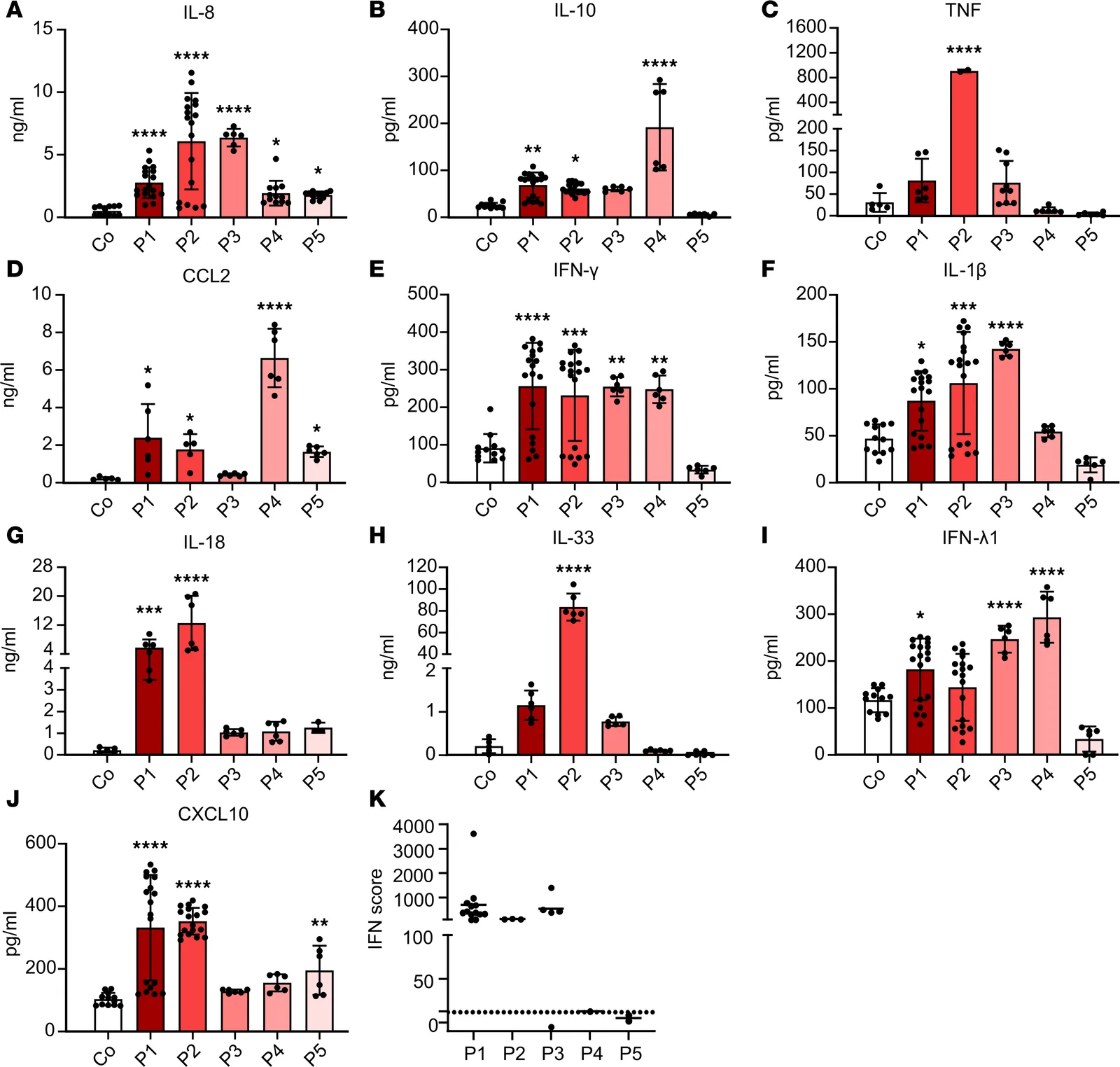
Increased secretion of proinflammatory cytokines in *RELA*-perturbed patient cells. (**A**–**J**) Serum levels of IL-8, IL-10, TNF, CCL2, IFN-γ, IL-1β, IL-18, IL-33, IFN-λ1, and CXCL10, collected at different time points, from patients (P1–P5) compared with 4 healthy controls (Co). Data are plotted as mean ± SD, *P < 0.05; **P < 0.01; ***P < 0.001; ****P < 0.0001 versus mean of WT controls, Kruskal-Wallis test (Dunn’s multiple-comparison test) for IL-8, CCL2, and IL-18 and 1-way ANOVA (Dunnett’s multiple-comparison test) for IL-10, TNF, IFN-γ, IL-1β, IL-33, IFN-λ1, and CXCL10. (**K**) IFN scores based on expression of IFN-stimulated genes in PBMCs. An IFN score of 12.49 (dashed line) indicates the median IFN score of 10 healthy controls + 2 SD.

Consistent with clinical signs of autoinflammation and autoimmunity, cytokine profiling of patient sera revealed elevated levels of a broad range of proinflammatory cytokines, including IL-8, IL-10, TNF, MCP-1 (CCL2), and IFN-γ compared with healthy controls (Figure 2, A–E). Notably, the inflammasome-dependent cytokines IL-1β and IL-18 (P1–P3) and the Th2-dependent cytokine IL-33 (P2) were also markedly elevated at baseline (Figure 2, F–H). In addition, all analyzed patients showed evidence of constitutive type I IFN activation, as shown by increased serum levels of IFN-λ (P1, P3, and P4), elevation of the type I IFN–dependent chemokine CXCL10 (P1, P2, P5) (Figure 2, I and J), or an increased blood IFN signature (P1–P3) (Figure 2K), in line with previous reports (11).

**Figure 3:**
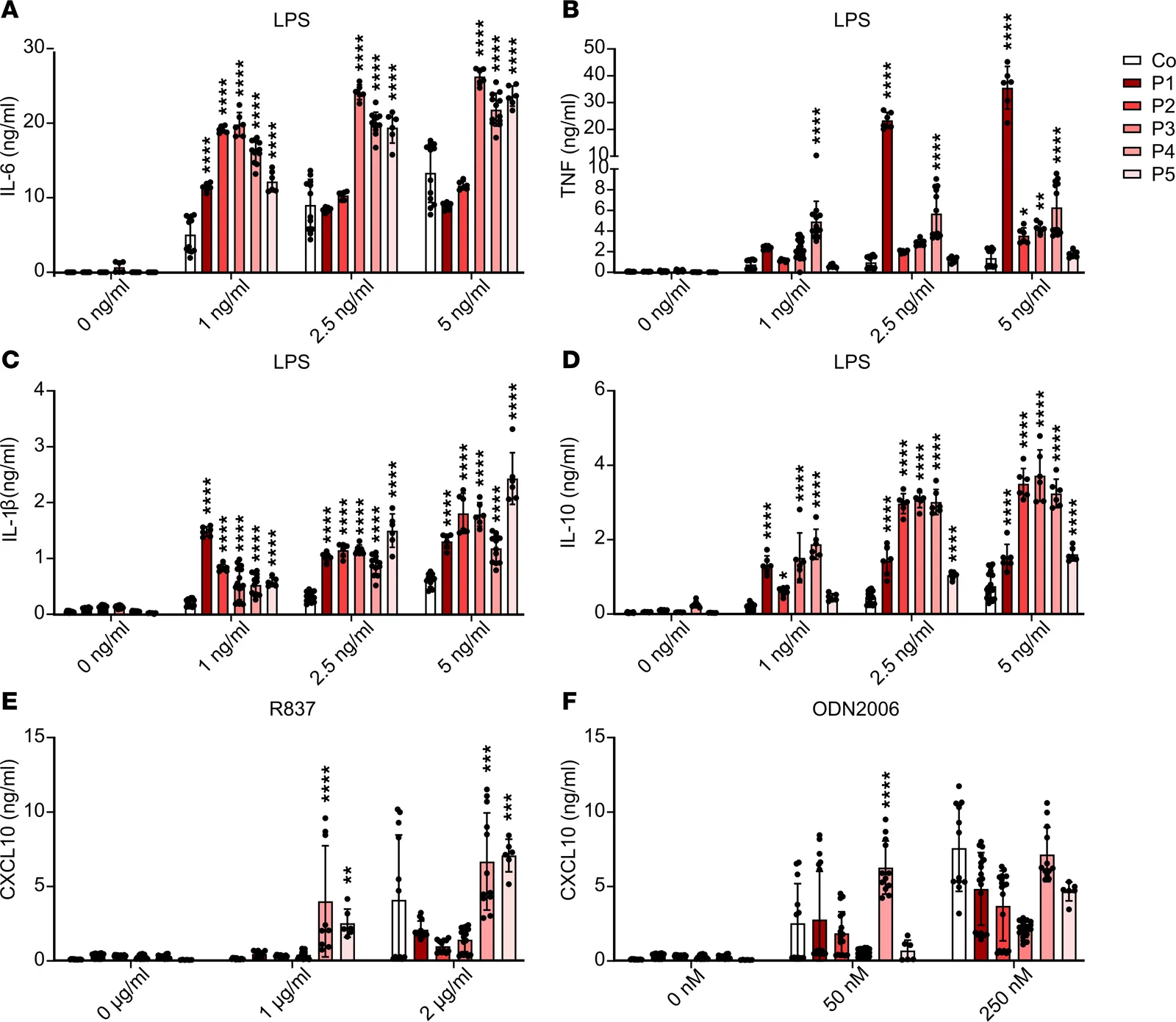
Enhanced cytokine responses after stimulation. (**A**–**D**) Secretion of IL-6, TNF, IL-1β, and IL-10 by whole blood from patients (P1–P5) after 24-hour stimulation with increasing concentrations of the TLR4 agonist LPS compared with 4 healthy controls (Co). Mean ± SD of 2 to 3 independent experiments. *P < 0.05; **P < 0.01; ****P < 0.0001 versus mean of WT controls, 2-way ANOVA (Dunnett’s multiple-comparison test). (**E** and **F**) Secretion of CXCL10 by whole blood from patients after 24-hour stimulation with increasing doses of the TLR7 agonist R837 or the TLR9 agonist ODN2006 compared with 4 healthy controls. Mean ± SD of 2 to 3 independent experiments. **P < 0.01; ***P < 0.001; ****P < 0.0001 versus mean of WT controls, 2-way ANOVA (Dunnett’s multiple-comparison test).

To further assess induced cytokine responses, we conducted whole-blood stimulation assays using different immunostimulatory ligands. After stimulation with increasing doses of the TLR4 agonist LPS, all examined patients (P1–P5) secreted significantly higher amounts of IL-6 at the lowest dose of 1 ng/mL, consistent with hypersensitivity to LPS, whereas at higher doses of 2.5 and 5 ng/mL, patients P3–P5 showed increased IL-6 secretion (Figure 3A). Similarly, TNF secretion was significantly increased by LPS stimulation in a dose-dependent manner in P1–P4 (Figure 3B). Notably, all patients also secreted higher amounts of IL-1β in response to LPS compared with controls, indicating hypersensitivity to inflammasome activation (Figure 3C). In addition, all patients showed increased IL-10 secretion upon LPS stimulation (Figure 3D). Similar to blood cells, primary fibroblasts from P1 were hyperresponsive to TNF stimulation compared with a WT control, as evidenced by a greater induction of the *IL6* gene (Supplemental Figure 2A). We next examined the patients’ response to stimulation of the type I IFN–inducing nucleic acid sensors TLR7 and TLR9, which have recently been shown to be hyperreactive in patients with truncating *RELA* mutations (11). Upon stimulation of whole blood with increasing doses of the TLR7 agonist R837, 2 (P4, P5) out of 5 patients responded with increased secretion of CXCL10, whereas CXCL10 secretion from patients P1, P2, and P3 did not differ from WT controls (Figure 3E). Similarly, after stimulation with the TLR9 agonist ODN2006, only P4 showed an increased CXCL10 response compared with WT controls (Figure 3F). Thus, in our cohort, constitutive type I IFN activation observed in all patients was not consistently associated with TLR7 and TLR9 hypersensitivity. Collectively, these findings demonstrate a global activation of multiple inflammatory pathways in patients with truncating *RELA* mutations that is accompanied by hyperresponsiveness to stimulation with LPS and TNF, and, to a lesser extent, to stimulation of TLR7 and TLR9.

### Altered canonical NF-κB signaling in patient cells.

**Figure 4:**
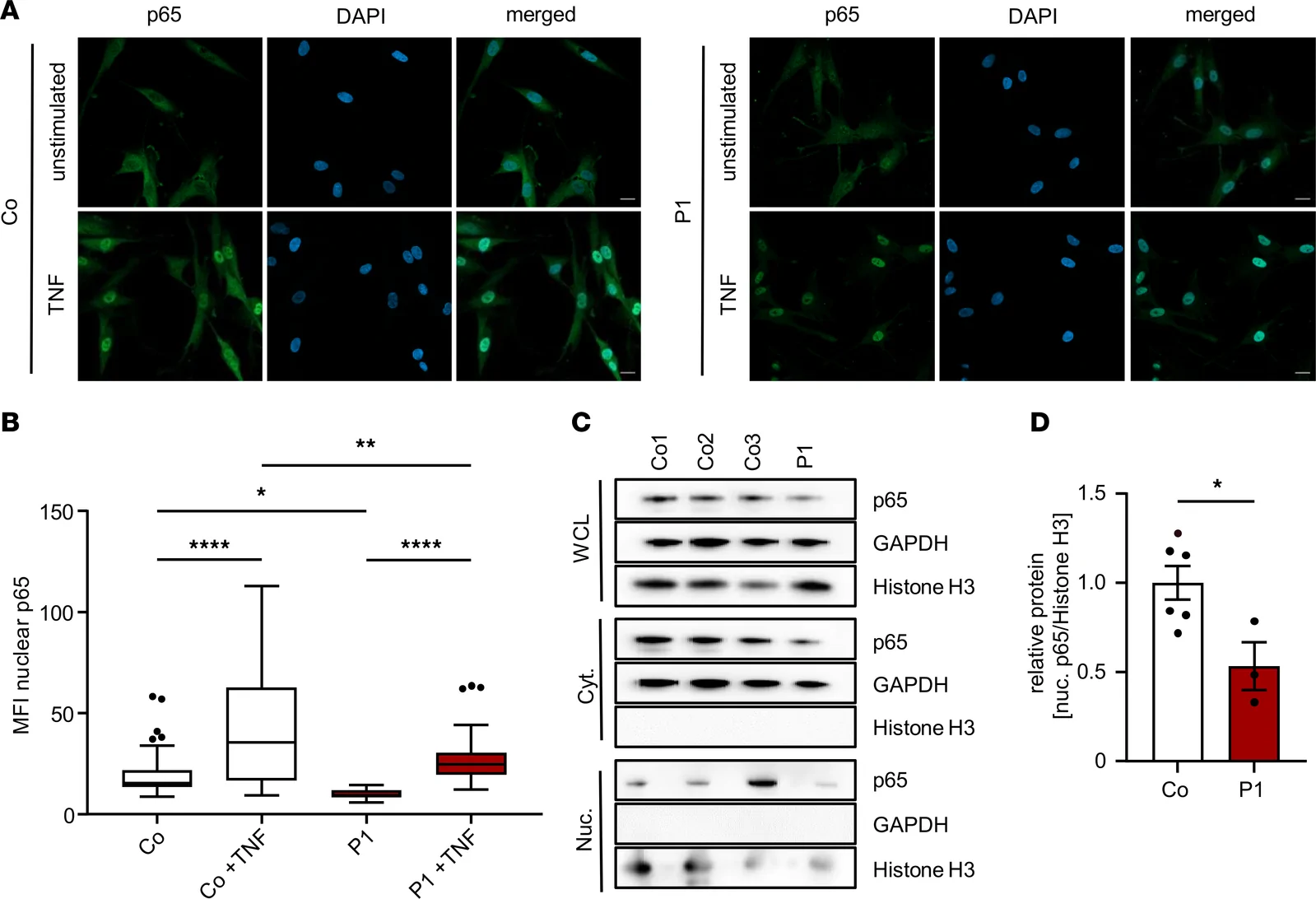
Altered nuclear translocation of p65 in patient fibroblasts. (**A**) Representative images of fibroblasts from patient and control immunostained for p65 (green) without and after TNF stimulation (10 ng/mL, 1 hour). Nuclei were stained with DAPI. Scale bars: 20 μm. (**B**) Quantitative analysis of nuclear p65 intensity. MFI in patient cells (*n* = 30–35) versus WT control cells (*n* = 28–32). Data presented as Tukey’s box plots with the box indicating the IQR, the center line indicating the median, and whiskers extending to 1.5 × IQR. *P < 0.05; **P < 0.01; ****P < 0.0001 versus mean of WT controls. One-way ANOVA (Šídák’s multiple-comparison test) was used for statistical analyses. (**C**) Representative immunoblot of p65 expression in whole cell lysate (WCL) and cytosolic (Cyt.) and nuclear fractions (Nuc.) of fibroblasts from P1 and 3 WT controls (Co1–3) after subcellular fractionation. GAPDH (cytoplasmic) and Histone H3 (nuclear) were used as loading controls. (**D**) Quantification of relative nuclear p65 in patient fibroblasts (P1) compared with WT controls, normalized to Histone H3. Mean ± SEM of at least 3 independent experiments. *P < 0.05 versus controls, Mann-Whitney *U* test.

The finding of upregulated proinflammatory cytokines in patient blood, both at steady state and in response to stimuli utilizing the canonical NF-κB pathway, is surprising given the inability of mutant p65 to perform its core function of dimer formation and gene transcription activation (Supplemental Figure 1, B and C). To investigate this further, we first examined the subcellular levels of p65 in patient cells. To distinguish WT from mutant p65, we used an antibody that recognizes the C-terminus and therefore only WT p65. Confocal imaging revealed reduced levels of cytosolic and nuclear WT p65 in P1 compared with the control (Figure 4, A and B), both in unstimulated cells and after TNF stimulation, consistent with reduced WT p65 dosage. An approximately 50% decrease of WT p65 in mutant cells was confirmed by Western blot analysis of cytosolic and nuclear fractions (Figure 4, C and D).

**Figure 5:**
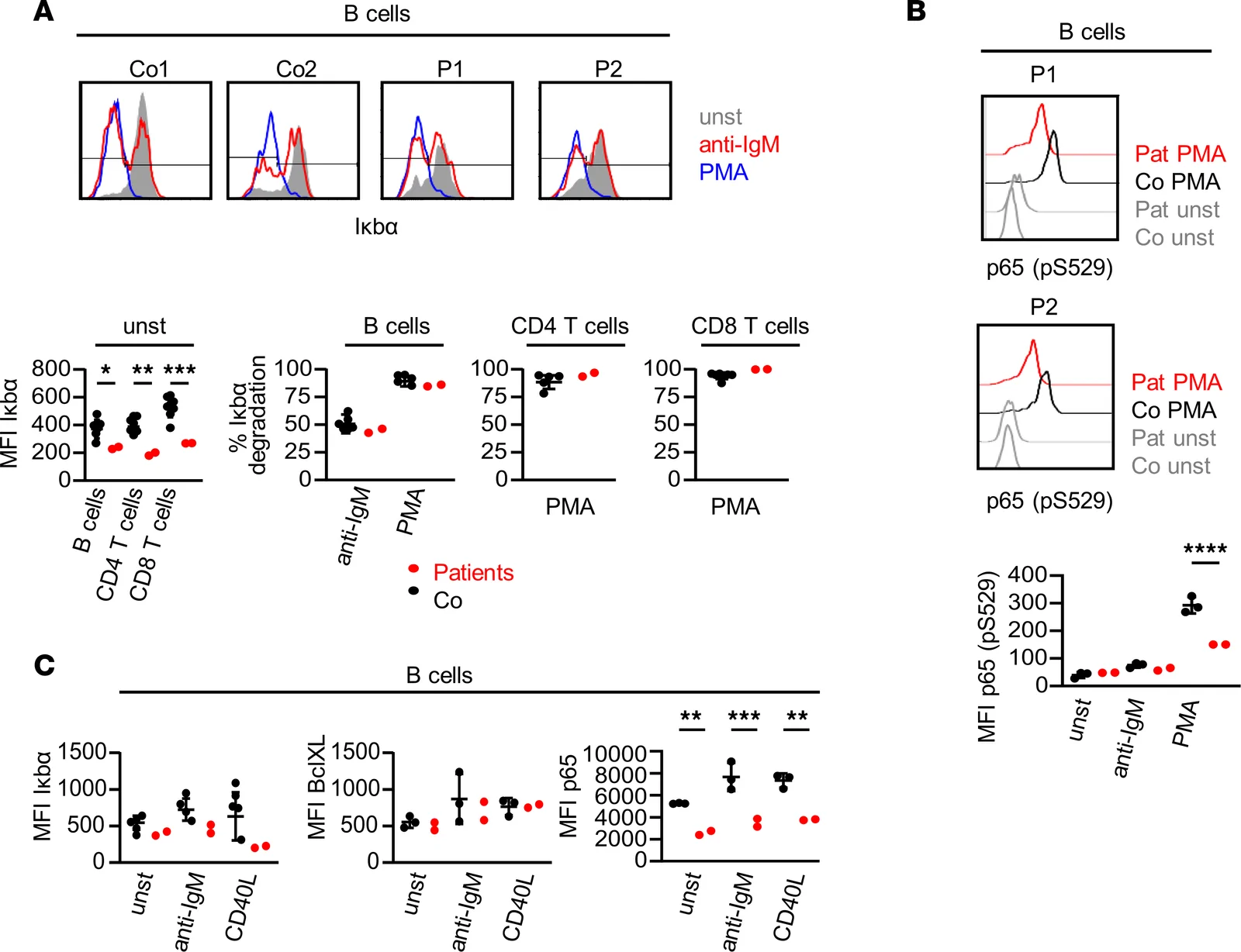
Altered NF-κB signaling in patient lymphocytes. (**A**) IκBα degradation in primary naive B cells stimulated with anti-IgM and PMA, and in T cells stimulated with PMA, of P1 and P2. IκBα protein levels at baseline and after stimulation in naive B, CD4^+^, and CD8^+^ T cells compared with healthy controls (Co). One-way ANOVA (Šídák’s multiple-comparison test); *P < 0.05; **P < 0.01; ***P < 0.001 versus mean of WT controls. (**B**) MFI of phosphorylated p65 upon activation with PMA in naive B cells of P1 and P2 compared with healthy controls. One-way ANOVA (Šídák’s multiple-comparison test); ****P < 0.0001 versus mean of WT controls. (**C**) Protein levels of p65 and its targets IκBα (*NFKBIA*) and BclXL (*BCL2L1*) after stimulation of PBMCs of P1 and P2 with anti-IgM and CD40L for 36 hours. One-way ANOVA (Šídák′s multiple-comparison test); **P < 0.01; ***P < 0.001 versus mean of WT controls.

We next analyzed canonical NF-κB signaling in lymphocytes from patients P1 and P2 by flow cytometry. We first examined the degradation of the inhibitory IκBα protein as the initial step in the signaling cascade leading to the release of the activated NF-κB dimer into the nucleus. Remarkably, in the unstimulated condition, IκBα protein, which is tightly regulated by the canonical NF-κB pathway, was lower in naive B, CD4^+^, and CD8^+^ T cells from both patients compared with healthy controls (Figure 5A). After stimulation of naive B cells with anti-IgM and PMA and of naive CD4^+^ and CD8^+^ T cells with PMA, respectively, the degradation of IκBα measured at 20 minutes after stimulation did not differ between patients and healthy controls (Figure 5A and Supplemental Figure 3A), suggesting normal stimulus-induced initiation of canonical NF-κB signaling. We then measured the activation-induced phosphorylation of p65. Consistent with the observed lower levels of p65 in patient cells at basal state (Figure 4, A and B), the levels of phosphorylated p65 (pSer529) were also lower compared with healthy controls in both naive B cells stimulated with anti-IgM or PMA and naive CD4^+^ and CD8^+^ T cells stimulated with PMA (Figure 5B and Supplemental Figure 3B). Thus, although basal expression of p65 was reduced, its phosphorylation in response to stimulation occurred normally. The normal IκBα degradation kinetics were consistent with the reduced but intact TNF-induced nuclear translocation of WT p65 observed by confocal microscopy at 30 minutes after stimulation (Figure 4, A and B).

To assess the effects of canonical NF-κB pathway stimulation on downstream targets, we stimulated PBMCs with anti-IgM and CD40L and then measured the protein levels of IκBα (NFKBIA) and Bcl-XL (BCL2L1) in naive B cells after 36 hours. Notably, although IκBα protein levels were adequately induced in B cells from healthy controls, they remained low in patient cells (Figure 5C and Supplemental Figure 3C). In contrast, poststimulation induction of Bcl-XL, which acts as an antiapoptotic protein, did not differ between patient cells and controls (Figure 5C and Supplemental Figure 3C). Consistent with the *RELA* haploinsufficiency, p65 protein levels in both unstimulated and stimulated cells were greatly reduced in patient B cells (Figure 5C and Supplemental Figure 3C). Taken together, these results indicate that activation of canonical NF-κB signaling upstream of p65 occurs normally in patient cells, albeit at lower levels. However, p65 activation was not accompanied by adequate poststimulation induction of IκBα, which could impair NF-κB inhibition at steady state and result in imbalanced NF-κB signaling.

### Impaired negative feedback control of NF-κB in patient cells and RELA gene-edited HEK293T cells.

**Figure 6:**
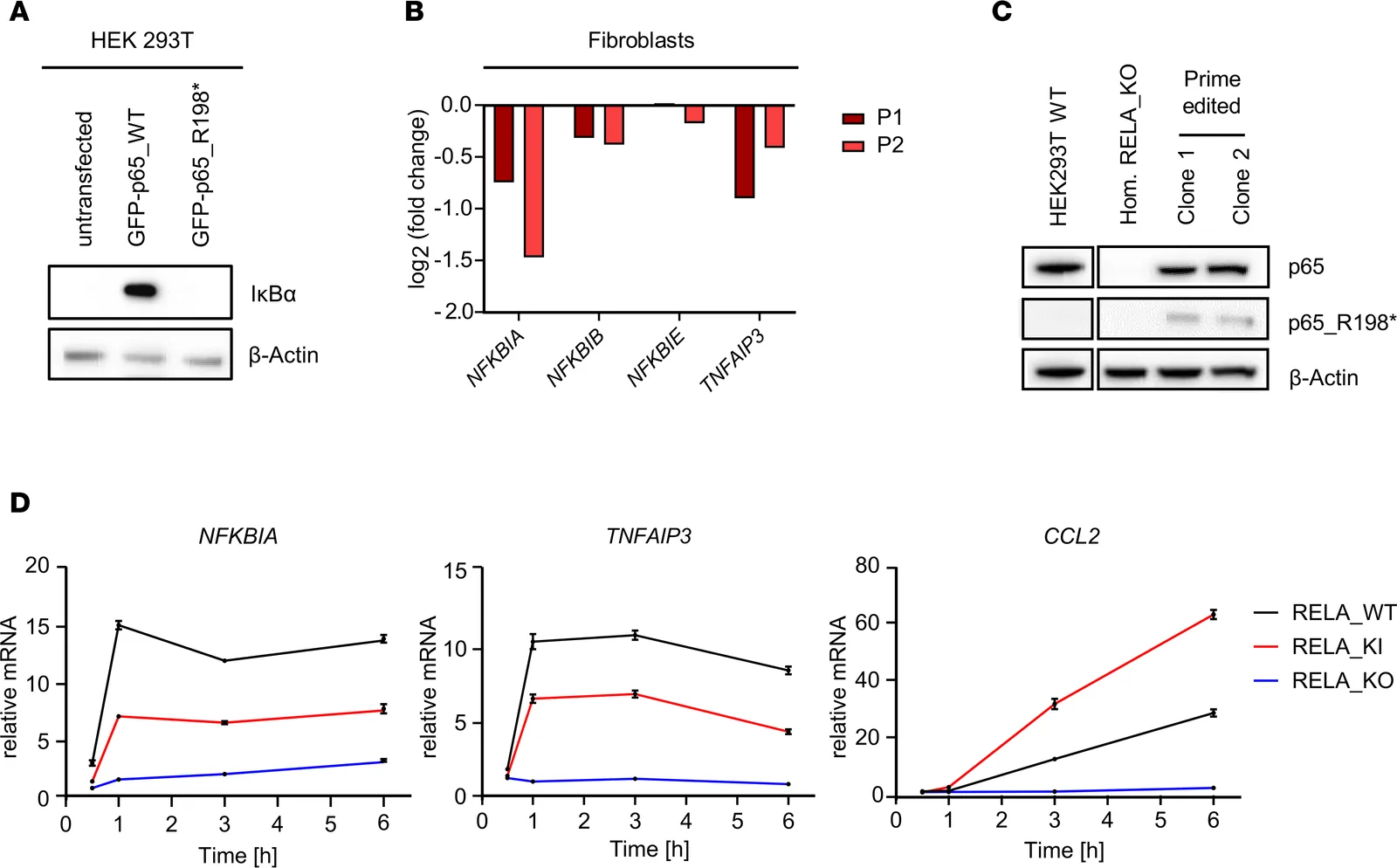
Impaired negative feedback control of NF-κB in patient cells and *RELA*^R198*^ KI cells. (**A**) IκBα protein expression in HEK293T cells transfected with either GFP-tagged WT p65 (GFP-p65_WT) or mutant p65^R198*^ (GFP-p65_R198*); β-actin was probed as loading control. (**B**) Differential gene expression of NF-κB inhibitors *NFKBIA* (IκBα), *NFKBIB* (IκBβ), *NFKBIE* (IκBε), and *TNFAIP3* (A20) in patient fibroblasts (P1, P2), shown as log_2_ fold-change compared with 3 WT controls. (**C**) Representative immunoblot of p65 expression in WT HEK293T cells (HEK293T WT) compared with homozygous *RELA* KO (RELA_KO) and 2 independent heterozygous *RELA*^R198*^ KI (RELA_KI) clones (clones 1 and 2). (**D**) Relative expression of *NFKBIA*, *TNFAIP3*, and *CCL2* in WT HEK293T cells (RELA_WT), *RELA* KO (RELA_KO), and *RELA*^R198*^ KI cells (RELA_KI) after stimulation with 100 ng/mL TNF for indicated time periods. Representative data from 1 out of 3 independent experiments run in triplicates. Data are plotted as mean ± SEM.

The transcriptional activity of NF-κB is tightly repressed by IκB proteins, including IκBα, IκBβ, and IκBε, through the formation of stable IκB/NF-κB complexes (2). After stimulus-induced IκB degradation, one of the earliest NF-κB transcriptional targets to be re-expressed is IκBα, providing negative feedback control (14). Consistent with this IκBα-dependent negative feedback mechanism, transfection of WT p65 into HEK293T cells resulted in a strong induction of IκBα (Figure 6A). In contrast, transfection of mutant p65^R198*^ had no effect on IκBα expression (Figure 6A), in agreement with its inability to transactivate a reporter gene (Supplemental Figure 1C).

Notably, at steady state, IκBα levels also strongly influence basal NF-κB activity by controlling the levels of nuclear NF-κB, which actively shuttles between the nucleus and cytosol. Although free IκBα is intrinsically unstable and rapidly degraded in an IKK-independent manner, NF-κB–bound IκBα is more stable (15). Thus, besides regulating its transcription, NF-κB also determines the fate of its own inhibitor IκBα through stabilization. Given the inability of mutant p65 to induce IκBα in HEK293T cells (Figure 6A), we reasoned that reduced steady-state levels of p65 might affect basal transcription of IκB protein-coding genes. We therefore examined the consequences of reduced WT p65 dosage on the expression of the *NFKBIA* (IκBα), *NFKBIB* (IκBβ), and *NFKBIE* (IκBε) genes in fibroblasts from P1 and P2. RNA-Seq revealed a marked decrease in the expression of all 3 NF-κB inhibitors in patient fibroblasts compared with 3 WT controls (Figure 6B), mirroring the reduced basal levels of IκBα observed in unstimulated patient lymphocytes (Figure 5A). Moreover, gene expression of the NF-κB inhibitor *TNFAIP3* was also reduced in patient fibroblasts (Figure 6B). Thus, at steady state, loss of a functional *RELA* allele in patient cells causes a shift in the balance between NF-κB activating and inhibitory factors toward activation.

To further investigate the functional consequences of the reduction of NF-κB inhibitory factors at the cellular level, we turned to a reductionist approach. We generated HEK293T cells (RELA_WT) with either a complete homozygous KO of the *RELA* gene (RELA_KO) or with a heterozygous KI of the R198* mutation at the *RELA* locus (RELA_KI) by CRISPR/Cas or prime editing, respectively (Figure 6C). Introduction of the R198* *RELA* mutation into HEK293T cells had no effect on cytokine gene expression in unstimulated cells (Supplemental Figure 4A). After stimulation of cells with 100 ng/mL of TNF, expression of the NF-κB inhibitory gene *NFKBIA* was strongly induced within 60 minutes in a *RELA* gene dose-dependent manner. Thus, the highest transcriptional activation of *NFKBIA* was seen in RELA_WT cells and the lowest in RELA_KO cells; RELA_KI cells showed an intermediate transcriptional response (Figure 6D), confirming the observation made in patient lymphocytes (Figure 5A) that a lack of *RELA* gene dose results in a correspondingly reduced induction of its inhibitor IκBα. Consistent with complete absence of functional p65 in RELA_KO cells, TNF stimulation had essentially no effect on the expression of *TNFAIP3* and *CCL2*, 2 bona fide NF-κB target genes (Figure 6D). Normally, TNF-induced expression of *TNFAIP3*, which functions as a key negative regulator of NF-κB signaling, peaks within 1 hour with a kinetic similar to *NFKBIA* (16). However, in contrast to RELA_WT cells, the induction of *TNFAIP3* was substantially reduced in RELA_KI cells, reflecting a weaker transactivation potential exerted by a single *RELA* allele (Figure 6D). Interestingly, in RELA_KI cells, in which the induction of the inhibitory genes *NFKBIA* and *TNFAIP3* was only half that of RELA_WT cells, the proinflammatory *CCL2* gene was strongly induced by more than 2.5-fold at 3 hours and more than 2.2-fold at 6 hours compared with RELA_WT cells (Figure 6D). Collectively, these findings suggest that the heightened proinflammatory response of RELA_KI cells to TNF stimulation may be due to a lack of inhibitory control of the NF-κB pathway.

### Upregulation of cell type–specific proinflammatory and cell death pathways in patient cells.

**Figure 7:**
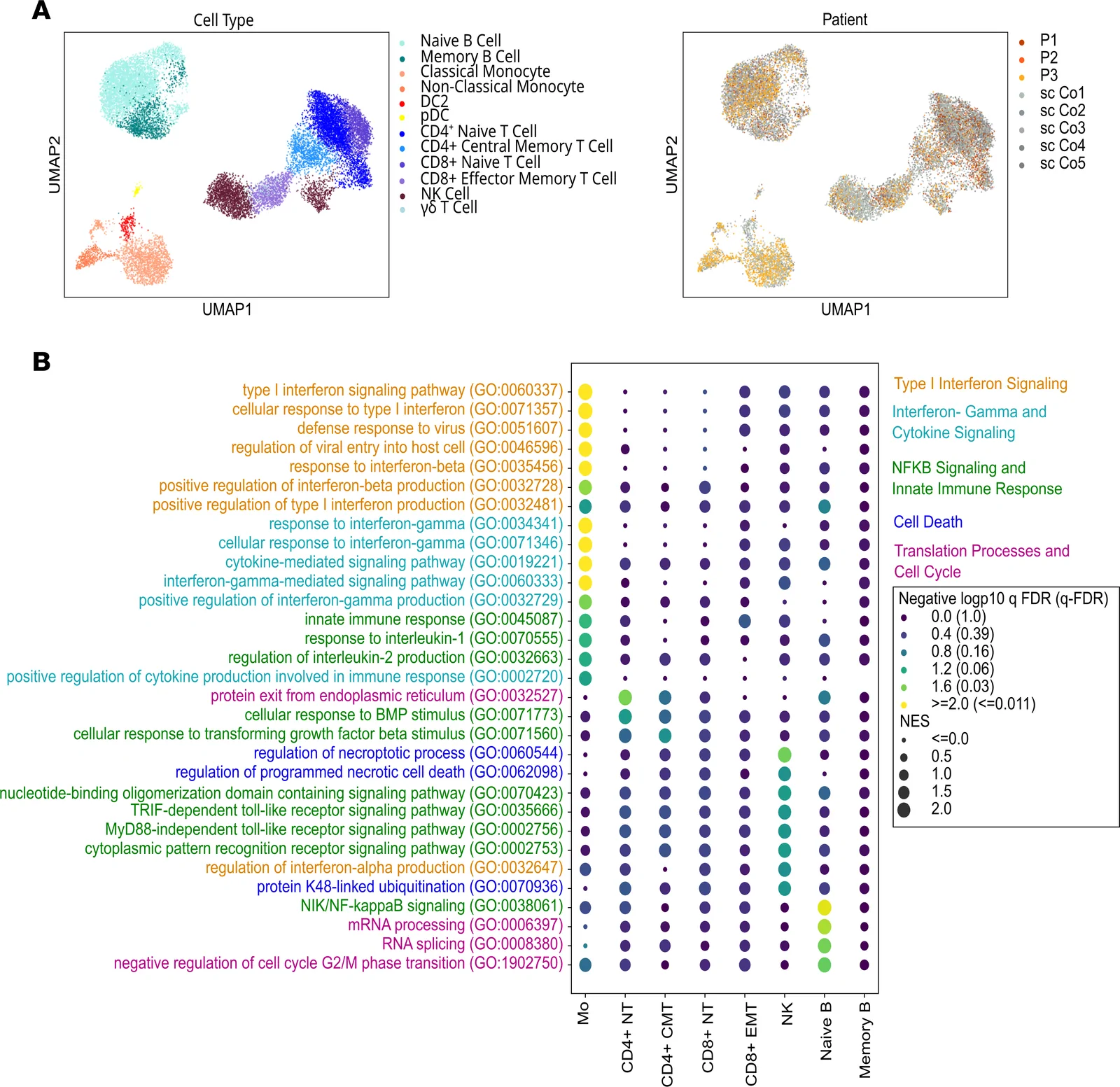
Cell type–specific transcriptomic alterations in *RELA*-perturbed patient cells. (**A**) Batch-corrected UMAP embedding of all cells profiled with scRNA-Seq from patient (P1–P3) and control (sc Co1–sc Co5) PBMCs. (**B**) GSEA enrichment of selected GO Biological Processes terms in differentially expressed genes between patient and control cells of the indicated cell types. For monocytes, only P3 and 2 controls (sc Co1, sc Co2) could be compared due to lack of monocytes in the other samples (see Methods). NES, normalized enrichment score.

To further investigate the transcriptional changes related to truncating *RELA* mutations in a cell type–resolved, untargeted manner, we performed single-cell RNA-Seq (scRNA-Seq) in PBMCs from P1, P2, and P3 along with 5 sex- and age-matched healthy controls. We identified and annotated all major cell types (naive and memory B cells, naive and central memory CD4^+^ and CD8^+^ T cells, γδ T cells, NK cells, classical and nonclassical monocytes, DCs, and plasmacytoid DCs [pDCs]) using annotation transfer and marker-based curation (Figure 7A and Supplemental Figure 5A). In line with clinical and laboratory data (Figure 2K), cells from all 3 patients expressed IFN-stimulated genes more highly than control cells — a signal mainly driven by myeloid cells but also present to a lesser degree in lymphoid cells (Supplemental Figure 5B). Cell type composition varied strongly, with no consistent differences between patient and control samples (Supplemental Figure 5C).

**Figure 8:**
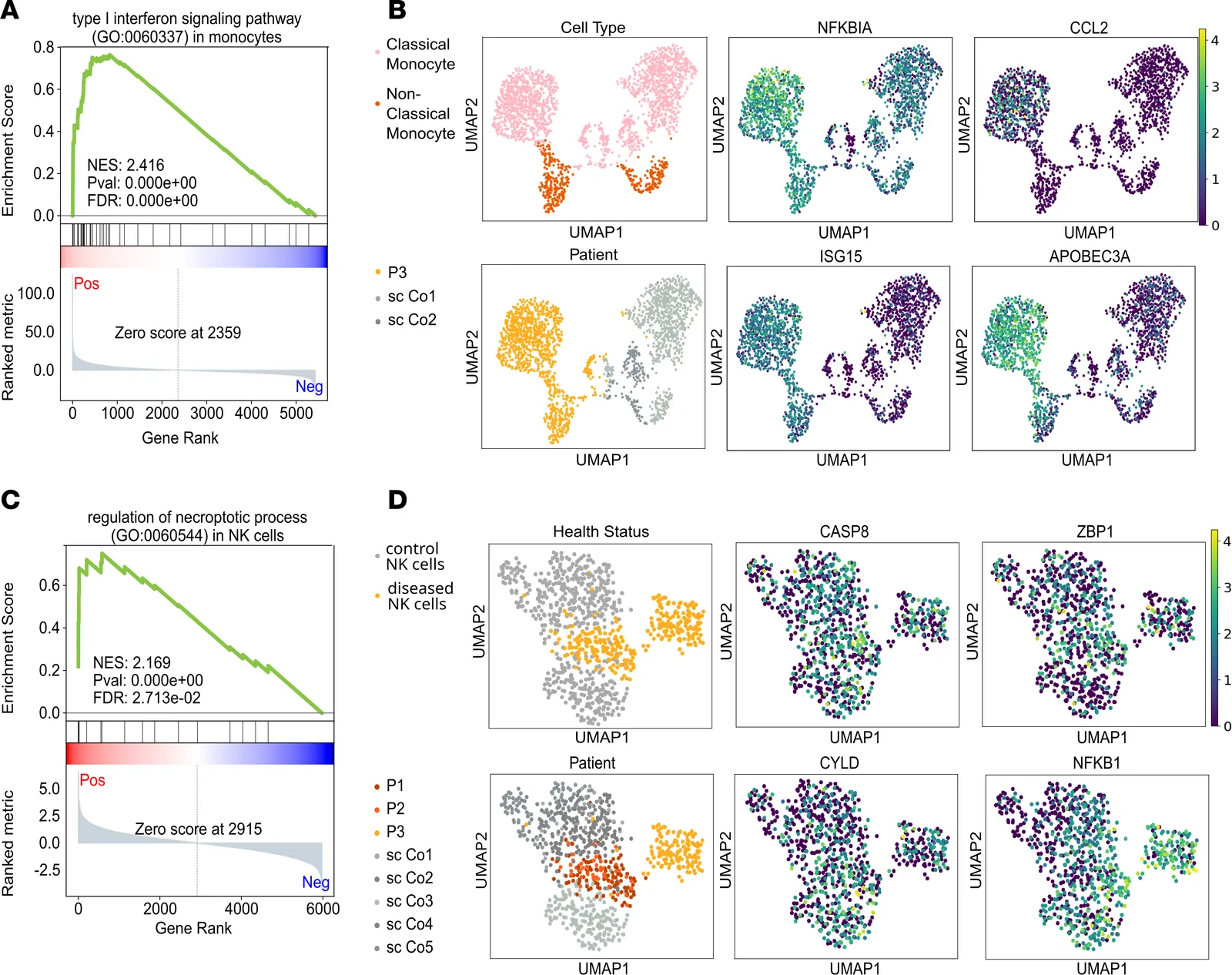
Activation of inflammatory and cell death pathways in *RELA*-perturbed patient cells. (**A**) GSEA leading-edge plot for the GO term type I IFN signaling pathway enriched in monocytes from P3 and control (sc Co1 and sc Co2) PBMCs. (**B**) UMAP embedding of monocytes colored by the sample assignment or the expression of the indicated genes in individual cells. (**C**) GSEA leading-edge plot for the GO term “regulation of necroptotic process” enriched in NK cells from patient (P1–P3) and control (sc Co1–sc Co5) PBMCs. (**D**) UMAP embedding of NK cells colored by the sample assignment or the expression of the indicated genes in individual cells.

Consistent with the hyperinflammatory phenotype observed in patients, gene set enrichment analysis (GSEA) revealed strong activation of multiple pathways related to proinflammatory cytokine production and signaling, including type I IFN, IFN-γ, and IL-1, particularly in monocytes (Figure 7B). Among the top-ranked gene sets enriched in monocytes were type I IFN signaling pathway (Gene Ontology [GO] 0060337) and response to IFN-γ (GO 0034341) (Figure 7B and Figure 8A). The IFN-stimulated genes *ISG15* and *APOBEC3G*, as well as the antiviral factor *BST2*, which can amplify proinflammatory responses via NF-κB signaling, showed increased and relatively homogeneous expression in both classical and nonclassical monocytes (Figure 8B and Supplemental Figure 5D). In contrast, the proinflammatory cytokine *CCL2* and *NFKBIA*, both NF-κB target genes, were more prominently upregulated in classical monocytes (Figure 8B), potentially indicating more WT-like NF-κB activity in classical monocytes.

In naive B cells from patients, NIK/NF-κB signaling (GO 0038061) emerged as the most highly enriched pathway (Figure 7B), suggesting a negative regulatory crosstalk of p65 on the alternative NF-κB signaling pathway in circulating B cells. Transcription factor activity analysis, considering the expression of all known target genes for a given transcription factor, further revealed elevated activity scores for *STAT1*, *NFKB*, and *RELA* in patient B cells (Supplemental Figure 6A). Patient B cells also showed elevated expression of the NF-κB inhibitors and target genes (*NFKBIA*, *NFKBID*, and *NFKBIE*), unlike patient T cells (Supplemental Figure 6B), providing evidence of constitutive NF-κB pathway activation in B cells.

In contrast, patient T and NK cells, in which IκBα protein levels were more strongly reduced than in B cells (Figure 5A), showed upregulation of cell death pathways, including both apoptosis and necroptosis (Figure 7B, Figure 8C, and Supplemental Figure 6C), the latter being a lytic, proinflammatory form of programmed cell death. This signature was most pronounced in NK cells (Figure 7B and Figure 8C). Apoptosis as an enriched term was also evident in naive CD4^+^ and CD8^+^ T cells but not in monocytes or B cells (Supplemental Figure 6C). Interestingly, the genes driving the apoptosis signal in T cells and NK cells showed substantial overlap with each other, but were largely distinct from those in monocytes (Supplemental Figure 6D). Notably, *CASP8* and *ZBP1* emerged among the most upregulated differentially expressed genes in NK cells (Supplemental Figure 6E) but still showed heterogeneous expression among patient and control cells (Figure 8D). *CASP8* encodes caspase-8, a critical initiator of apoptosis downstream of death receptor signaling; ZBP1 (Z-DNA binding protein 1) functions as a cytosolic nucleic acid sensor that can activate both necroptosis and apoptosis. Through these mechanisms, ZBP1 contributes to inflammation via the release of danger-associated molecular patterns (DAMPs) (17, 18). Accordingly, the highest ranked gene set enriched in patient NK cells was regulation of necroptotic process (GO 0060544) (Figure 7B). This enrichment was among others driven by increased expression of *CASP8*, *ZBP1*, and *CYLD*, and *JUN* was also among the top differentially expressed genes (Figure 8D, Supplemental Figure 5D, and Supplemental Figure 6E), consistent with chronic activation of both apoptotic and necroptotic cell death pathways. In addition, transcription factor activity analysis revealed the highest activity scores for *JUN*, *ATF4*, and *CREB1* in CD4^+^ naive T cells from patients (Supplemental Figure 6A), reflecting a state of activation and stress-associated dysfunction. Jun is associated with T cell activation, promotes Th1 and Th17 differentiation, and has been linked to T cell exhaustion (19). ATF4 acts as a central mediator of the integrated stress response and regulates the expression of proinflammatory cytokines under cellular stress conditions (20). CREB1 contributes to the transcription of key cytokines such as IL-2 (essential for T cell proliferation); IFN-γ (critical for Th1 responses); and under certain conditions, IL-17 (involved in Th17 differentiation) (21). Together, these transcriptional signatures support a bias toward proinflammatory effector T cells, primarily Th1 and Th17, which are known to play key roles in initiating and sustaining chronic inflammation. Overall, the cell type–resolved transcriptional comparison of patient versus control immune cells uncovered a differential signature marked by reduced NF-κB inhibition and heightened apoptotic/necroptotic signaling, particularly in T and NK cells.

To verify that these findings are unique to *RELA*-mutated patients and not a broader feature of inflammatory processes, we analyzed publicly available scRNA-Seq data from SLE and COVID-19 cohorts using the same pipeline (Supplemental Figure 7). Genes previously highlighted as differentially expressed in patient monocytes and NK cells, such as *APOBEC3A*, *ISG15*, and *JUN* (Figure 8D, Supplemental Figure 5D, and Supplemental Figure 6E), showed no significant upregulation in the corresponding cell types for either SLE or COVID-19 (Supplemental Figure 7, C and D). GSEA confirmed heightened inflammatory signatures in SLE and COVID-19, as expected. Similarly, pathways central to our interpretation of the *RELA* mutation were not perturbed in the same way in the SLE and COVID-19 cohorts. Notably, necroptosis-related pathways were not enriched in COVID-19 at all and were restricted to T cells in SLE, whereas in our data, these signatures were prominently enriched only in NK cells (Supplemental Figure 7E). These comparisons support a disease-specific transcriptional program of *RELA* mutations compared with other inflammatory diseases.

### Enhanced sensitivity to TNF-induced apoptosis and necroptosis in patient cells and RELA gene-edited HEK293T cells.

Upon TNF receptor engagement, adaptor proteins including RIPK1 and cIAP1/2 assemble complex I, a membrane-associated platform where ubiquitinated RIPK1 activates NF-κB and promotes survival (22). Deubiquitination of RIPK1 by CYLD, A20, or OTULIN shifts signaling toward cytosolic complex II, composed of FADD, procaspase-8, and RIPK1, where caspase-8 activation initiates apoptosis and simultaneously suppresses necroptosis by cleaving RIPK1 and RIPK3. In the absence or inhibition of caspase-8, RIPK1 and RIPK3 interact via RHIM domains to form the necrosome, which drives inflammatory necroptosis, particularly in response to TNF (23).

**Figure 9:**
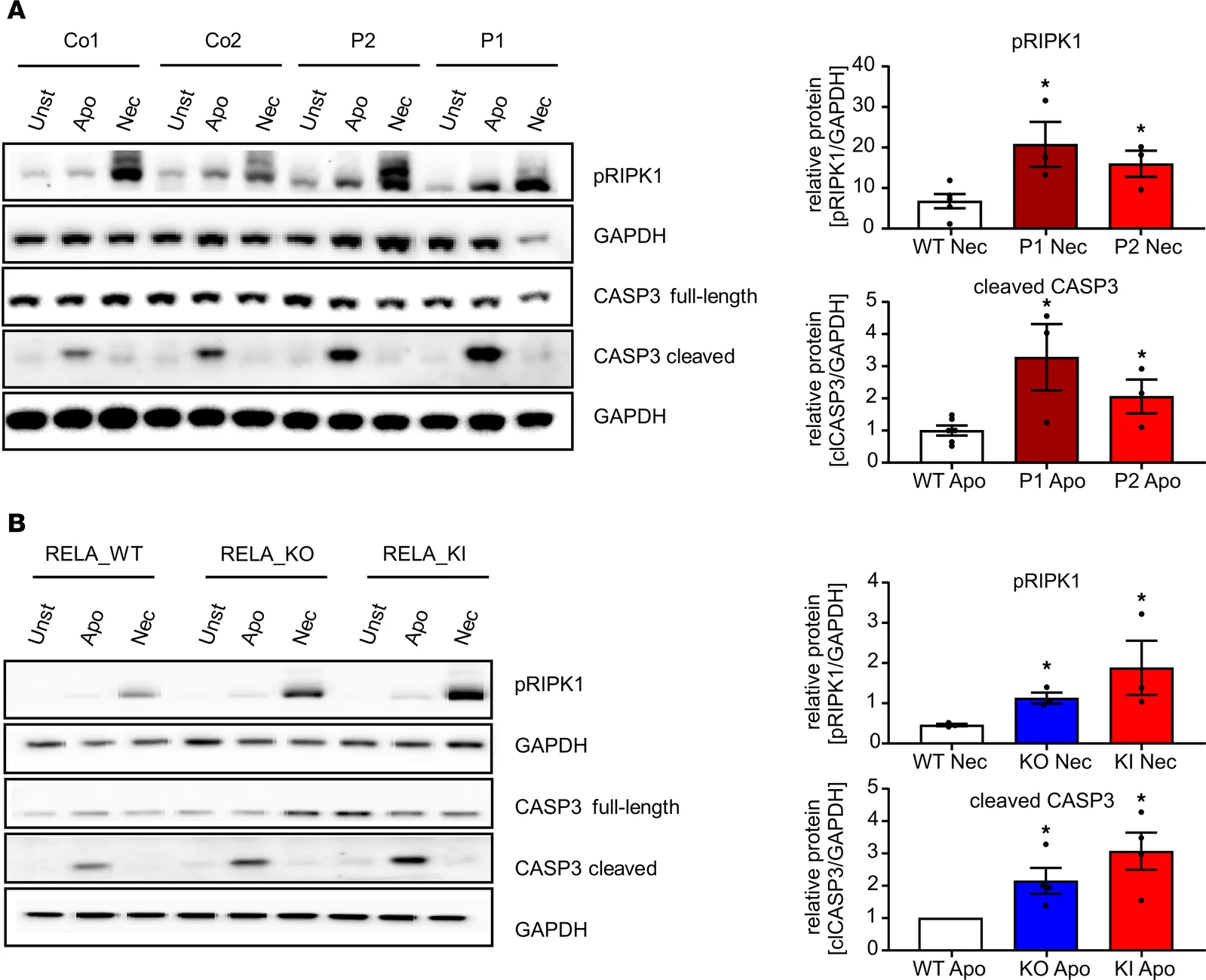
Increased sensitivity to apoptosis and necroptosis conferred by the *RELA*^R198*^ mutation. (**A**) Left: representative immunoblots showing increased phosphorylated RIPK1 (pRIPK1; Ser166) and cleaved caspase-3 (CASP3) in patient fibroblasts (P1, P2) versus WT controls (Co1, Co2). GAPDH was stained as loading control. Right: quantification of 3 independent experiments (ImageJ [NIH], normalized to GAPDH). *P < 0.05 versus WT (Mann-Whitney *U* test; mean ± SEM). (**B**) Left: representative immunoblots showing increased pRIPK1 and cleaved CASP3 in RELA_WT, RELA_KO, RELA_KI HEK293T cells. GAPDH was probed as loading control. Right: quantification of 3 to 4 independent experiments (ImageJ [NIH], normalized to GAPDH). *P < 0.05 versus theoretical mean = 1 (1-sample *t* test; mean ± SEM). Apo: TNF + birinapant; Nec: TNF + birinapant + emricasan.

Given the increased apoptosis/necroptosis signaling (Figure 7B and Supplemental Figure 6C); upregulation of *CASP8* and *CYLD* (Figure 8D and Supplemental Figure 6, D and E); and a reduction in negative regulation of NF-κB signaling (Figure 5A), observed predominantly in patient T and NK cells, we hypothesized that patient cells may be intrinsically more sensitive to cell death. To test this, we stimulated patient cells with TNF in the presence of the SMAC mimetic birinapant, which inhibits cIAPs and promotes apoptosis (24). In a parallel condition, cells were stimulated with TNF and cotreated with birinapant and the pan-caspase inhibitor emricasan to block apoptosis and enforce necroptosis (25). Upon treatment of fibroblasts from P1 and P2 with birinapant, TNF stimulation led to a stronger induction of cleaved caspase-3, indicating enhanced apoptosis compared with WT controls (Figure 9A). Similarly, cotreatment with birinapant and emricasan resulted in markedly increased phosphorylation of RIPK1 at Ser166, a hallmark of necroptosis activation, compared with WT controls, suggesting heightened necroptotic potential in patient cells (Figure 9A). Notably, RELA_KI cells, expressing only 1 functional *RELA* allele, also exhibited increased apoptosis and necroptosis in response to TNF stimulation compared with RELA_WT or RELA_KO cells (Figure 9B), confirming that *RELA* haploinsufficiency underlies the heightened sensitivity to TNF-induced cell death observed in patient cells. Densitometric quantification of all immunoblots in patient fibroblasts and RELA_WT/KO/KI HEK293T cells confirmed significantly increased levels of cleaved caspase-3 under apoptotic conditions (Figure 9A) and phosphorylated RIPK1 under necroptotic conditions (Figure 9B). Collectively, these findings demonstrate that the loss of a functional *RELA* allele compromises TNF tolerance and predisposes cells to inflammatory cell death.

## Discussion

This study delineates the clinical, immunological, and mechanistic consequences of heterozygous truncating mutations in *RELA* encoding the NF-κB subunit p65. By combined analysis of patient-derived cells and gene-edited heterologous cell models, we demonstrate that these variants confer *RELA* haploinsufficiency. Gene-edited HEK293T cells served as a reductionist isogenic system to isolate the direct consequences of *RELA* haploinsufficiency on NF-κB feedback control and TNF-induced cell death and served to exclude secondary systemic effects due to heightened immune activation (Figures 6 and 9), but their nonimmune nature limited their ability to model immune cell responses. However, all key mechanistic findings were independently validated in primary patient-derived lymphocytes and fibroblasts, ensuring physiological relevance. This state of reduced gene dosage impairs the negative regulatory control of NF-κB signaling, leading to insufficient induction of inhibitory feedback regulators such as IκBα and A20. As a consequence, the tightly regulated interplay between the pro-survival and pro-death functions of NF-κB becomes compromised, shifting immune homeostasis toward immune dysregulation and ultimately driving self-sustaining hyperinflammation.

We identified 4 truncating *RELA* mutations, which to our knowledge were previously unreported, in 6 patients presenting with a spectrum of autoinflammatory and autoimmune disease of varying severity. All patients exhibited inflammatory skin disease, including Behçet-like mucosal ulcerations, erythema nodosum, erythroderma, and malar rash. Five out of 6 patients showed systemic autoinflammatory features, such as recurrent fever and elevated inflammatory markers, and 4 patients developed intestinal inflammation, consistent with previous reports of *RELA*-associated disease (5–8, 11–13). Autoimmune manifestations such as vitiligo, hypothyroidism, autoimmune anemia, and the presence of autoantibodies further highlight the dual autoinflammatory-autoimmune phenotype associated with truncating *RELA* variants.

All identified mutations were either inherited in an autosomal dominant manner or occurred de novo and disrupted the transactivation domain of p65 required for transcriptional activity. Consistent with this, the R198* mutant failed to form homodimers or p65/p50 heterodimers, abolishing its core functional capacity. Remarkably, P3, who carried the most C-terminal truncating mutation reported to date (A507Pfs*25), exhibited a clinical phenotype highly similar in both symptoms and severity to that of P1 with the more N-terminal R198* mutation. Previous reports have described dominant-negative *RELA* variants associated with type I interferonopathy (11), but we did not observe variant-specific clinical or cellular signatures in P3 or P4 that would support such a mechanism. Rather, all truncating variants in our cohort are consistent with haploinsufficiency. This indicates that impairment of the transactivation domain alone is sufficient to confer a loss-of-function phenotype.

Mechanistically, *RELA* haploinsufficiency primarily impairs negative feedback regulation rather than upstream NF-κB activation. Despite preserved stimulus-induced phosphorylation of p65, patient cells failed to adequately induce IκBα and other inhibitory regulators such as A20, resulting in impaired termination of NF-κB signaling. RELA^R198*^ KI HEK293T cells recapitulated this phenotype, showing reduced induction of inhibitory regulators but enhanced and prolonged expression of proinflammatory cytokines such as CCL2. Importantly, these amplified inflammatory responses depended on the residual activity of the functional *RELA* allele and not on the mutant protein, underscoring a gene-dosage effect in which 1 functional *RELA* allele seems to be sufficient to activate inflammatory target genes but insufficient to maintain proper negative feedback control of NF-κB signaling.

Although prompt activation of NF-κB is essential for host defense, uncontrolled NF-κB signaling can be detrimental and impede immune homeostasis, promoting chronic inflammation. Among its negative regulatory factors, IκB proteins play a central role in limiting the intensity and duration of NF-κB responses (14–16). In our patients, p65 levels were consistently reduced, in line with *RELA* haploinsufficiency, and basal IκBα levels differed across cell types. With otherwise preserved stimulus-induced NF-κB activation, insufficient induction of IκBα and A20 emerged as the critical defect disturbing the negative feedback loop that normally constrains NF-κB activity at steady state.

Single-cell transcriptomics revealed that the consequences of this impairment were cell type specific: Monocytes showed strong enrichment of type I IFN, IFN-γ, and IL-1 signaling pathways, with increased expression of *ISG15*, *APOBEC3G*, and *BST2*. B cells exhibited enhanced *RELA* activity and alternative NF-κB signaling, which warrants further investigation but may reflect compensatory mechanisms supporting survival and antibody function (26).

By contrast, T cells and NK cells, which exhibited markedly reduced IκBα levels, were skewed toward apoptotic and necroptotic programs, characterized by increased expression of *CASP8*, *ZBP1*, and *CYLD*, alongside increased activity of stress-related transcription factors *JUN*, *ATF4*, and *CREB1*. This transcriptional landscape indicates that *RELA* insufficiency renders lymphocytes particularly vulnerable to stress-induced and inflammatory cell death, thereby fueling chronic inflammation through the release of DAMPs (27). To assess disease specificity, we compared our *RELA*-specific results with publicly available scRNA-Seq datasets from SLE and COVID-19 cohorts. Although inflammatory signatures were present throughout, as expected, key features of patients with *RELA* mutations, such as the NK cell–specific enrichment of necroptosis-related pathways, were not observed. These findings suggest a disease-specific transcriptional program, though the lack of a fully matched inflammation control cohort remains a study limitation. Consistently, patient fibroblasts and RELA_KI cells demonstrated enhanced apoptosis and necroptosis upon TNF stimulation, reflecting heightened susceptibility to inflammatory cell death. These findings are consistent with prior work in *RELA*-deficient mice and fibroblasts, which demonstrated profound TNF sensitivity and tissue injury (5, 28, 29). Notably, although complete p65 deficiency in mice causes embryonic lethality due to extensive hepatocyte apoptosis, heterozygous *RELA*-KO mice remain phenotypically normal (30). The discrepancy between heterozygous *RELA* deficiency in mice and the pronounced phenotype in humans may reflect differences in microbial and inflammatory exposures, which constantly challenge the NF-κB regulatory capacity in humans but not in mice maintained under pathogen-free conditions. The marked clinical variability among patients may similarly reflect differences in environmental triggers that aberrantly activate NF-κB signaling.

In conclusion, we demonstrate that truncating *RELA* mutations impair NF-κB feedback control, resulting in heightened inflammatory signaling, TNF sensitivity, and increased apoptotic and necroptotic cell death. This mechanistic framework explains the diverse clinical features observed in patients with truncating *RELA* mutations and suggests new therapeutic avenues aimed at restoring NF-κB feedback regulation and limiting inflammatory cell death.

## Methods

### Sex as a biological variable.

The patients in our study were male and female; sex was not considered as a biological variable. However, when comparing patients with healthy controls, samples were matched for sex and age.

### Exome sequencing.

Exome sequencing was performed as part of the routine diagnostic workup for patients with suspected monogenic immune dysregulation. Genomic DNA was isolated from peripheral blood leukocytes, and whole-exome sequencing was carried out in accredited diagnostic laboratories following standard protocols. Sequence data were aligned to the human reference genome (GRCh37/hg19 or GRCh38, depending on the center) and analyzed for rare protein-altering variants in genes associated with immune-related disorders. Candidate variants were confirmed by Sanger sequencing and assessed for segregation within families when parental samples were available. Identified variants were classified according to the guidelines of the American College of Medical Genetics and Genomics.

### Sanger sequencing.

Genomic DNA sequences flanking *RELA* (NM_021975.4) gene mutations were amplified by PCR using gene-specific primers (Eurofins MWG Operon) and sequenced in both directions using the BigDye Terminator v1.1 Cycle Sequencing kit (Thermo Fisher Scientific, 4337449) on a 3130xl Genetic Analyzer (Applied Biosystems). Data were analyzed using Vector NTI software (Life Technologies).

### Cell culture and stimulation.

Primary fibroblasts obtained from patients and healthy controls (passages 4 to 20) and HEK293T cells were cultured in DMEM (Sigma-Aldrich, D6546) supplemented with 2 mM L-glutamine (Gibco, 25030-024), 1% antibiotics/antimycotics (Gibco, 15240-062), 5% nonessential amino acids (NEAA, Gibco, 11140-035) and 10% FBS (Sigma-Aldrich, S0615) at 37°C under 5% CO_2_. Cells were treated with 10 or 100 ng/mL TNF (Prospec Protein Specialists, 112PTNFA31). For isolation of human PBMCs, whole blood diluted with PBS was gently layered over an equal volume of BioColl (Sigma-Aldrich) and centrifuged for 30 minutes at 400*g* without brake. The intermediate layer containing PBMCs was removed and added to prewarmed medium.

### Mutagenesis.

For mutagenesis, the GFP-RelA plasmid (Addgene, 23255) was used. The mutations (R198*, S169*, A507Pfs*25, Q372*) were introduced by site-directed mutagenesis using QuikChange Lightning (Agilent Technologies, 210518) or Q5 Site-Directed Mutagenesis kit (NEB, E0554S) and the following primers (Eurofins Genomics): R198*: fwd: CGAGCTCAAGATCTGCTGAGTGAACCGAAACTC and rev: GAGTTTCGGTTCACTCAGC-AATCTTGAGCTCG. S169*: fwd: CGGGACCCATGAGGCAGGCCC and rev: CACTGTCACCTGGAAGCAGAGCC. A507Pfs*25: fwd: GGGCCCAGAGGCCCC and rev: GTCACTAGGCGAGTTATAGCCTCAGG. Q372*: fwd: TCCTTCTGGGTAGATCAGCCA and rev: AACACCATGGTGGGAAAC.

### Western blot analysis.

The cells were collected and washed twice with PBS. Pellets were lysed in RIPA buffer (50 mM TRIS-HCl, pH 7.4, 150 mM NaCl, 1 mM EDTA, 1% Triton X-100, 1 mM sodium orthovanadate, and 20 mM sodium fluoride) supplemented with 1× Complete Protease Inhibitor Cocktail, 1× PhosSTOP phosphatase inhibitors (Roche), and 1 U/mL DNase I (Qiagen, 79254). Protein concentration was determined using a BCA kit (Thermo Fisher Scientific). Lysates were resolved in 4% to 12% NuPAGE Bis-TRIS gel (Thermo Fisher Scientific) under reducing and denaturing conditions and blotted onto a nitrocellulose membrane (Sigma-Aldrich). Membranes were blocked in 5% nonfat dry milk (AppliChem, APPA0830,0500) or in 5% BSA (Sigma-Aldrich, 05470) in 1× TBS plus 0.1% Tween-20 (SERVA, 37470.01) and incubated overnight at 4°C using the following antibodies: anti-GAPDH (Cell Signaling Technology, 2118), anti-p65 (Santa Cruz, sc-8008), anti-IkBα (R&D Systems, AF4299), anti-p50 (Thermo Fisher Scientific, MA5-15870), anti-Histone H3 (Cell Signaling Technology, 9715S), anti-β-actin (Sigma-Aldrich, A5316), anti-pRIPK1 (Cell Signaling, 65746), anti-CASP3 (Cell Signaling Technology, 9668), and anti-GFP (Cell Signaling Technology, 2555S). Antibodies were diluted in 5% dry milk or 5% BSA in 1× TBS plus 0.1% Tween-20. Immunoreactive signals were detected by chemiluminescence using the SuperSignal West Femto Maximum Sensitivity Substrate (Thermo Fisher Scientific, 34095) on an Azure imaging system. Quantification of imaged bands was performed using ImageJ (NIH).

### Co-IP.

For co-IP, HEK293T cells were lysed in RIPA buffer supplemented with 1× Complete Protease Inhibitor Cocktail, 1× PhosSTOP phosphatase inhibitors (Roche), and 1 U/mL DNase I. Lysates were incubated with equilibrated G-agarose beads (Thermo Fisher Scientific, 53125) for 1.5 hours at 4°C and then centrifuged at 2500*g* and 4°C for 5 minutes. The supernatant was removed, and after 3 washing steps with 500 μL ice-cold PBS and renewed centrifugation, precipitated proteins were eluted and denatured in 80 μL 2× SDS loading buffer, incubated at 95°C for 10 minutes, and centrifuged (2500*g* at 4°C for 2 minutes). The supernatant removed corresponded to the eluate. SDS-PAGE and Western blotting of the eluted protein samples and signal detection were performed as described above.

### Bulk RNA-Seq.

RNA-Seq was performed as previously described (31).

### scRNA-Seq.

Frozen PBMCs from P1 (sc P1), P2 (sc P2), and P3 (sc P3), along with 5 age- and sex-matched controls (sc Co1, sc Co2, sc Co3, sc Co4, sc Co5), were rapidly thawed at 37°C and immediately resuspended in 5% FBS/PBS. After centrifugation at 350–550*g* for 5 minutes at 4°C, the cell pellets were resuspended and incubated for 10 minutes with 5 μL of 10% Human TruStain FcX Fc Receptor Blocking solution (BioLegend) in PBS. For sample multiplexing, 48.5 μL of antibody master mix (47.5 μL of 5% FBS/PBS, 0.5 μL of PE anti-human CD123, and 0.5 μL of APC anti-human CD303 [BDCA-2]) and 1.5 μL of TotalSeq-C antibody hashtags (BioLegend) were added to each sample. The samples were then incubated at 4°C for 20 minutes, followed by washing and resuspension in 5% FBS/PBS. Cell counts were assessed using the Invitrogen Countess II Automated Cell Counter, and samples were pooled to ensure equal cell contributions. Pool 1 consisted of P1 and P2 and controls sc Co3, sc Co4, and sc Co5 and 3 more samples not relevant to this study; pool 2 consisted of P3 and controls sc Co1 and sc Co2 and 5 more samples not relevant to this study. SYTOX Green (Thermo Fisher Scientific, S7020) diluted 1:1,000 in PBS was added, and dead cells were removed, while pDCs were enriched using the FACSAria III Cell Sorter (BD Biosciences). To focus on lymphoid cell populations, myeloid cells were filtered out for pool 1 and were retained for pool 2. After cell sorting, 2 populations were obtained: (a) pDCs and (b) remaining PBMCs. Both cell populations were centrifuged, and the pellets were resuspended in 0.1% BSA in PBS: pDCs in 300 μL and the remaining PBMCs in 100 μL. Cell concentration of the PBMC fraction was quantified using the Countess automated cell counter (Thermo Fisher Scientific). The cell concentration was recorded and based on the 10x Genomics’ recommended loading concentration (33,000 cells for a target recovery of 20,000), and the required volume of PBMCs to reach this target was calculated. The 300 μL pDC-only suspension was then divided equally into two 150 μL aliquots. The appropriate volume of sorted PBMCs, calculated to yield a total of 33,000 cells per reaction, was added to each aliquot to generate pDC-only aliquot to generate pDC-enriched cell suspensions. These mixed suspensions were subsequently spun down and the supernatant was carefully removed to leave 38.7 μL or less final volume, consistent with the maximum volume allowable for loading onto the 10x Genomics Chromium platform, and loaded onto 2 lanes of the Chip G of the Chromium Next GEM Single Cell 5′ v2 (Dual Index) kit (10x Genomics). GEX libraries were constructed according to the kit instructions. Both libraries were sequenced on a P3 Flow Cell on an Illumina NextSeq 1000 machine.

### scRNA data analysis.

Raw sequencing data in FASTQ format were processed to generate a gene-cell expression matrix using the Cell Ranger 7.1.0 count pipeline. The parameters were set with expect-cells = 20,000 and include-introns = True, utilizing the GRCh38-2020-A human genome reference. Cells were demultiplexed using an in-house hashtag demultiplexer and genetically with CellSNP-lite (v1.2.2) (32) and Vireo (v0.5.8) (33). Doublets were identified and subsequently removed using our hashtag demultiplexer Vireo (v0.5.8) (33) and scDblFinder (v1.8.0) (34). Further analysis was conducted using the Scanpy framework (v1.9.3) (35). Quality filtering was performed using pp.filter_cells(min_genes = 200) and pp.filter_genes(min_cells = 3). Cells with more than 25% of transcripts mapped to the mitochondrial genome were excluded. The filtered dataset consisted of 54,272 cells. Normalization to correct for library size was carried out using the pp.normalize_total(target_sum = 10,000) function, ensuring comparability of counts among cells. The normalized data underwent log-transformation using the pp.log1p function. Highly variable genes were identified using pp.highly_variable_genes and were utilized for dimensionality reduction and clustering. Principal component analysis (PCA) was performed using tl.pca. A neighborhood graph for cells was generated using the pp.neighbors function with n_neighbors = 15. The neighborhood graphs were then embedded using the tl.umap function and visualized through the pl.umap function. UMAPs depicting all cell types were batch-corrected for “processing batch” using harmony scanpy.external.pp.harmony_integrate (36). Cell-type annotation was done using model-based automatic cell-type annotation CellTypist (v1.5.0) (37) using the models Immune_all_Low.pkl and Immune_all_High.pkl. Annotation was further refined manually using established cell marker genes.

Differential gene expression analysis was done using decoupleR (v.1.6.0) (38) in a pseudo-bulk manner (except for monocytes). Pseudo-bulks were generated using dc.get_pseudobulk, which sums up the counts per cell type per patient. Genes were further filtered using dc.plot_filter_by_expr (group=’mutation’, min_count = 10, min_total_count = 15) and the in decoupleR integrated pydeseq2 (v.0.4.9) used for statistical tests. The DESeq2 object was built for each cell type using DeseqDataSet(refit_cooks = True, inference = DefaultInference) and as design_factors the variable denoting diseased or healthy cells given. To compute log fold-changes, dds.deseq2 was run and the contrast extracted with DeseqStats. For hypothesis testing, the Wald test was applied using contrast.summary. Transcription factor activity analysis was also done using decoupleR and their univariate linear model decoupler.mt.ulm with CollecTRI (39) as a resource for transcriptional regulatory interactions. Because monocytes were only collected from 1 patient (P1) and the 2 corresponding controls, a pseudo-bulk approach as above was not possible, and differential gene expression analysis was done in a single-cell manner using scanpy.tl.rank_genes_groups using method = *t*-test.

GSEA was performed using GSEApy (v1.1.4) (40), utilizing gene rankings derived from the differential gene expression analysis based on the *t* value. For monocytes, only genes expressed in at least 10% of the diseased cells were considered. For all other cell types, only genes with an average mean expression of at least 10 counts were included. The Human MSigDB Hallmark gene sets 2020 (41, 42) and the GO Biological Processes gene sets 2021 (43, 44) were tested for enrichment using the function prerank. The publicly available 5′ scRNA-Seq data of COVID-19 and SLE were processed and analyzed as described, together with their matched controls (45, 46).

### Luciferase-based reporter assay.

The following day, 500,000 cells/well of the NF-κB/293/GFP-Luc Transcriptional Reporter cell line (System Biosciences, TR860A-1) were seeded for transfection. The pEGFP-N1 (GenBank accession U55762), GFP-p65_WT, GFP-p65_R198*, GFP-p65_S169*, GFP-p65_A507Pfs*25, and GFP-p65_Q372* plasmids were transfected using lipofectamine 3000 (Invitrogen, L3000-008) reagent following the manufacturer’s manual. The luciferase assay system (Promega, E1500) was used for measurement of luciferase activity according to the manufacturer′s instructions. Bioluminescence was quantified using a Mithras LB 940 multiplate reader (Berthold Technologies).

### Immunofluorescence.

Cells were fixed with 4% formaldehyde, permeabilized with 0.25% Triton X-100 in PBS for 5 minutes at room temperature, and blocked with 1% BSA in PBS for 1 hour at room temperature before overnight incubation with the primary antibody (anti-p65; Invitrogen, 51-0500) diluted in 1% BSA-containing PBS at 4°C. After 3 washes with PBS, cells were incubated with the appropriate Alexa Fluor–labeled secondary antibody (Invitrogen, A-11008) diluted in 1% BSA-containing PBS for 1 hour in the dark at room temperature. Finally, cells were washed an additional 3 times with PBS before mounting with Vectashield-containing DAPI. Confocal microscopy was performed using an inverted LSM980 (Zeiss) equipped with an Airyscan detector unit. Images were acquired using a 20×/0.8 plan-apochromat objective with *Z*-stack series at 0.31 μm intervals for 7 sections using the piezo drive, followed by raw image processing using the Airyscan processing and extended depth-of-focus functions in Zen Black software (Zeiss). MFI was determined in individual cells using a custom-written MATLAB code (47).

### Apoptosis and necroptosis assay.

Primary human fibroblasts and HEK293T cells were cultured to confluence in DMEM complete medium. For apoptosis and necroptosis induction, TNF (BioLegend, 570108), birinapant (Biomol, Cay19699-5), and emricasan (Biomol, Cay22204-5) were dissolved in DMSO and a master mix was prepared using DMEM complete medium. Cells were exposed to 1 μM birinapant for 5 hours, followed by 20 ng/mL TNF for a further 14 hours to induce apoptosis. To induce necroptosis, cells were first incubated with 1 μM birinapant and 5 μM emricasan for 5 hours, followed by 20 ng/mL TNF for a further 14 hours. After stimulation, cells were trypsinized, centrifuged (800*g*, 5 minutes), and washed twice with PBS. For downstream analysis, pellets were stored at –80°C. DMSO was used as solvent control.

### Cell fractionation.

Frozen cell pellets from cultured fibroblasts were resuspended in ice-cold 0.1% NP40 in PBS. An aliquot of the lysate was removed as whole cell lysate, mixed with 6× Laemmli sample buffer, and then kept on ice until sonication. The remaining lysate was centrifuged and the supernatant removed as cytosolic fraction, mixed with 4× Laemmli sample buffer, and boiled at 95°C for 1 minute. The remaining pellet was resuspended in 1 mL of ice-cold 0.1% NP40 in PBS and centrifuged. After discarding the supernatant, the pellet was resuspended with 1× Laemmli sample buffer as the nuclear fraction. Whole cell lysate and nuclear fraction were sonicated for 5 seconds at a high level and then boiled for 1 minute at 95°C. Cell fractions were verified by Western blot analysis using anti-GAPDH and anti-Histone H3 as cytoplasmic and nuclear fraction–specific antibodies, respectively, as described (48).

### Quantitative RT-PCR.

RNA was extracted with the ReliaPrep RNA Cell Miniprep system (Promega, Z6012) followed by DNase I digestion. RNA was reverse-transcribed using the GoScript Reverse Transcription System (Promega, A5001). Target gene expression was determined by quantitative RT-PCR using GoTaq qPCR Master mix (Promega, A6002) on a QuantStudio 5 Real-Time PCR system (Applied Biosystems). Relative expression of mRNAs was analyzed by the Ct value of target genes and quantified by normalizing to *GAPDH* (fwd: GAAGGTGAAGGTCGGAGTC; rev: GAAGATGGTGATGGGATTTC) and hypoxanthine phosphoribosyltransferase 1 (fwd: AGATGGTCAAGGTCGCAAG; rev: TTCATTATAGT-CAAGGGCATATCC) using the ΔΔCt method. The following primers were used: *CXCL10* (fwd: GTGGCATTCAAGGAGTACCTC; rev: TGATGGCCTTCGATTCTGGATT); *MX1* (fwd: GTTTCCGAAGTGGACATCGCA; rev: CTGCACAGGTTGTTCTCAGC); *IL-6* (fwd: AGACAGCCACTCACCTCTTCAG; rev: TTCTGCCAGTGCCTCTTTGCTG); *TNFAIP3* (fwd: CTCAACTGGTGTCGAGAAGTCC; rev: TTCCTTGAGCGTGCTGAACAGC), *NFKBIA* (fwd: TCCACTCCATCCTGAAGGCTAC; rev: CAAGGACACCAAAAGCTCCACG); *CCL2* (fwd: AGAATCACCAGCAGCAAGTGTCC; rev: TCCTGAACCCACTTCTGCTTGG); *TNF* (fwd: ATGAGCACTGAAAGCATGATCC; rev: GAGGGCTGATTAGAGAGAGGTC).

### Blood IFN score.

Total RNA was extracted from PBMCs using the ReliaPrep RNA Cell Miniprep system (Promega, Z6012), followed by DNase I digestion. RNA was reverse-transcribed using the GoScript Reverse Transcription system (Promega, A5001). Gene expression was determined by quantitative RT-PCR using TaqMan Universal PCR Master Mix (Applied Biosystems, 4427788) on an ABI7300 and normalized to *GAPDH* (fwd: GAAGGTGAAGGTCGGAGTC; rev: GAAGATGGTGATGGGATTTC) and hypoxanthine phosphoribosyltransferase 1 (Hs02800695_m1, Thermo Fisher Scientific) expression. For calibration, a calibrator cDNA was included in each assay. Target genes were analyzed using predesigned TaqMan probes (Thermo Fisher Scientific) for *IFI27* (Hs01086373_g1), *IFI44* (Hs00951349_m1), *IFI44L* (Hs00915292_m1), *IFIT1* (Hs01675197_m1), *ISG15* (Hs01921425_s1), *RSAD2* (Hs01057264_m1), and *SIGLEC1* (Hs00988063_m1). The IFN score was calculated as previously described (49).

### Cytokine analysis.

Cytokines were quantified using the LEGENDplex Human Inflammation Panel 1 (BioLegend) and the LEGENDplex Human Anti-Virus Response Panel (BioLegend) according to the manufacturer’s instructions. Data were collected on an LSR Fortessa flow cytometer (BD Biosciences) and analyzed with LEGENDplex Data Analysis V8.1 software (BioLegend).

### Whole blood assay.

Heparinized blood was distributed in 140 μL aliquots into a 96-well plate and maintained in RPMI medium (Gibco, 31870-025) at 37°C. For analysis of induced cytokine responses, blood was stimulated with LPS (1, 2.5, or 5 ng/mL; InvivoGen, tlrl-b5lps), R837 (1 or 2 μg/mL; InvivoGen, tlrl-imqs-1), or ODN2006 (50 or 250 nM; InvivoGen, tlrl-2006) for 24 hours at 37°C. After incubation, plates were centrifuged at 800*g* for 5 minutes at room temperature. Supernatants were pipetted into a new 96-well plate and frozen at –80°C.

### Intracellular flow cytometry.

IκBα degradation and phosphorylation of p65 were determined as described previously with minor adaptations (50, 51). In brief, 3.5 × 10^5^ PBMCs were left untreated or stimulated with 15 μg/mL F(ab)′2 goat anti-human IgM (Southern Biotech) for 35 minutes, recombinant CD40L for 15 minutes or 50 ng/mL PMA (Sigma Aldrich) for 15 minutes. Cells were fixed by addition of Cytofix and permeabilized by using Perm III (both BD Biosciences) following the manufacturer′s instructions. After permeabilization, cells were stained with the appropriate antibodies to discriminate T and B cell subsets and measured on an LSR Fortessa (BD Biosciences). For measurement of IκBα, Bcl-xL, and p65, 3 × 10^5^ PBMCs were stimulated with 10 μg/mL anti-human IgM and CD40L for 36 hours. Zombie NIR (BioLegend) was added 10 minutes prior to fixation with Cytofix and permeabilization with Perm III as described above. After washing, cells were stained with the respective antibodies and measured on an LSR Fortessa. Data were analyzed with FlowJo 10.10. The following antibodies were used: anti-CD19 Brilliant Violet 421, anti-CD21 PE-Cy7, anti-CD38 PerCp-Cy5.5, anti-CD45RA BV605, anti-CD3 BV650, and anti-CD4 BV786 (all BioLegend); anti-CD27 Brilliant Violet 605, anti-CD27 BUV395, anti-CD8 BUV495, anti-p65(pS256) Alexa Fluor 488, and anti-IκBα PE, (all BD Biosciences); anti-Bcl-xL Alexa Fluor 488 and rabbit anti-human p65 XP AF647 (Cell Signaling Technology); anti-IgD FITC (Southern Biotech); and anti-IgM Alexa Fluor 647 (Jackson Immunoresearch Laboratories).

### Generation of RELA_KO and RELA_KI HEK293T cells.

For CRISPR/cas9-mediated generation of *RELA* KO cells, the px459 plasmid pSpCas9(BB)-2A-Puro (Addgene, 62988), containing a cutting Cas9, sgRNA scaffold, and a selection marker, was used for insertion of targeting sequences as described (52). sgRNA targeting the *RELA* gene was constructed using the following oligonucleotides (Eurofins Genomics): fwd: TAATACGACTCACTATAGTGCCGAGTGAACCGAA; rev: TTCTAGCTCT-AAAACGAGTTTCGGTTCACTCGGC. For insertion of the sgRNA into the plasmid, the following guides were used: fwd: CACCGTGCCGAGTGAACCGAAACTC; rev: AAACGAGTTTCGGTTCACTCGGCAC. HEK293T cells were seeded in 6-well plates and transfected with the Px459 plasmid including the inserted sgRNA scaffold using lipofectamine 3000 (Thermo Fischer Scientific) in DMEM complete media containing 10 mg/mL puromycin; 24 hours after transfection, medium was replaced with DMEM complete with 1 μg/mL puromycin. After completion of puromycin selection, cells were sorted into a 96-well plate for generation of single clones. For prime editing, a pU6-pegRNA-GG acceptor was used to generate heterozygous *RELA* R198* KI cells (RELA_KI). PegRNA templates were synthesized by PCR and then cloned into the pU6-pegRNA-GG-acceptor. Successful editing was verified by Sanger sequencing and by Western blot analysis using anti-p65.

### Statistics.

Statistical analysis was carried out in GraphPad Prism 10. For normally distributed variables, parametric tests were used, including a 2-tailed Student’s *t* test, 1-sample *t* test, or 1-way/2-way ANOVA, as appropriate. For variables with non-normal distribution, nonparametric tests were used. A *P* value less than 0.05 was considered statistically significant. Data are presented as mean ± SD or mean ± SEM, as indicated.

### Study approval.

The study was conducted in accordance with the Declaration of Helsinki and with approval by the ethics committee (EK386102017, BO-EK-466102022) of the Medical Faculty, Technische Universität Dresden. Written informed consent was obtained from the patients or their legal representatives.

### Data availability.

RNA-Seq data generated in this study have been deposited in the Single Cell Portal (accession SCP2151) and in NCBI’s Gene Expression Omnibus (GEO GSE339599) and are publicly available. All other data are available within the article or the Supporting Data Values file.

## Author contributions

NL and MALK conceptualized the research. MALK supervised clinical data curation and experimental research. JK supervised scRNA-Seq data generation and analysis. NL, S Weidler, BK, AD, SE, TS, MB, S Wagner, PS, S Koss, S Kretschmer, and CW conducted the experiments, acquired data, and analyzed results. AAE, TJSR, MM, and AK generated scRNA-Seq data. TBH, SVH, RAJ, and UH conducted exome sequencing and variant analysis. AAE performed scRNA-Seq data analysis with contributions from SWM. OS, LI, TN, MF, HO, OCB, CS, and JKD provided clinical data. JS acquired patient material. MALK wrote the manuscript with contributions from NL, S Weidler, CW, AAE, and JK. Among the co–first authors, the authorship order reflects the relative contribution to experimental work, data analysis, and manuscript preparation. All authors reviewed and edited the manuscript.

## Conflict of interest

JKD has received grant support, honoraria, and speaker fees from Novartis and SOBI.

## Funding support

- German Research Foundation (Deutsche Forschungsgemeinschaft; DFG) grants LU 2342/1-1 (to NL), CRC237 369799452/B21 (to MALK and JK), CRC237 369799452/A11 (to MALK), CRC369 501752319/C06 (to MALK), CRC237 369799452/A06 (to CW), and project 545533246 (to BK).
- Federal Ministry of Research, Technology and Space (Bundesministerium für Forschung, Technologie und Raumfahrt, BMFTR) grant 01GM2206C (GAIN, to MALK), 01GM2206A (GAIN, to BK), and as part of the German Center for Child and Adolescent Health (DZKJ) under the funding code 01GL2405B (to CS and MALK).
- European Union.
- Platform for Precision Medicine and Molecular Prevention, co-funded by the European Union and co-financed by tax revenues an the basis of the budget adopted by the Saxon State Parliament (PräMo, 100779691; to NL and MALK).
- DFG Gerok fellowship (CRC237 369799452) to S Weidler.
- Else Kröner-Fresenius Foundation (Else Kröner-Fresenius-Stiftung; EKFS) starting grant (2019_A70) to JK.
- German Academic Exchange Service (Deutscher Akademischer Austauschdienst e. V.; DAAD) PhD fellowship to MM.
- DFG (project 446167311) funded the LSR Fortessa.

## Supplementary Material

Supplemental data

Unedited blot and gel images

Supporting data values

## Figures and Tables

**Table 1:**
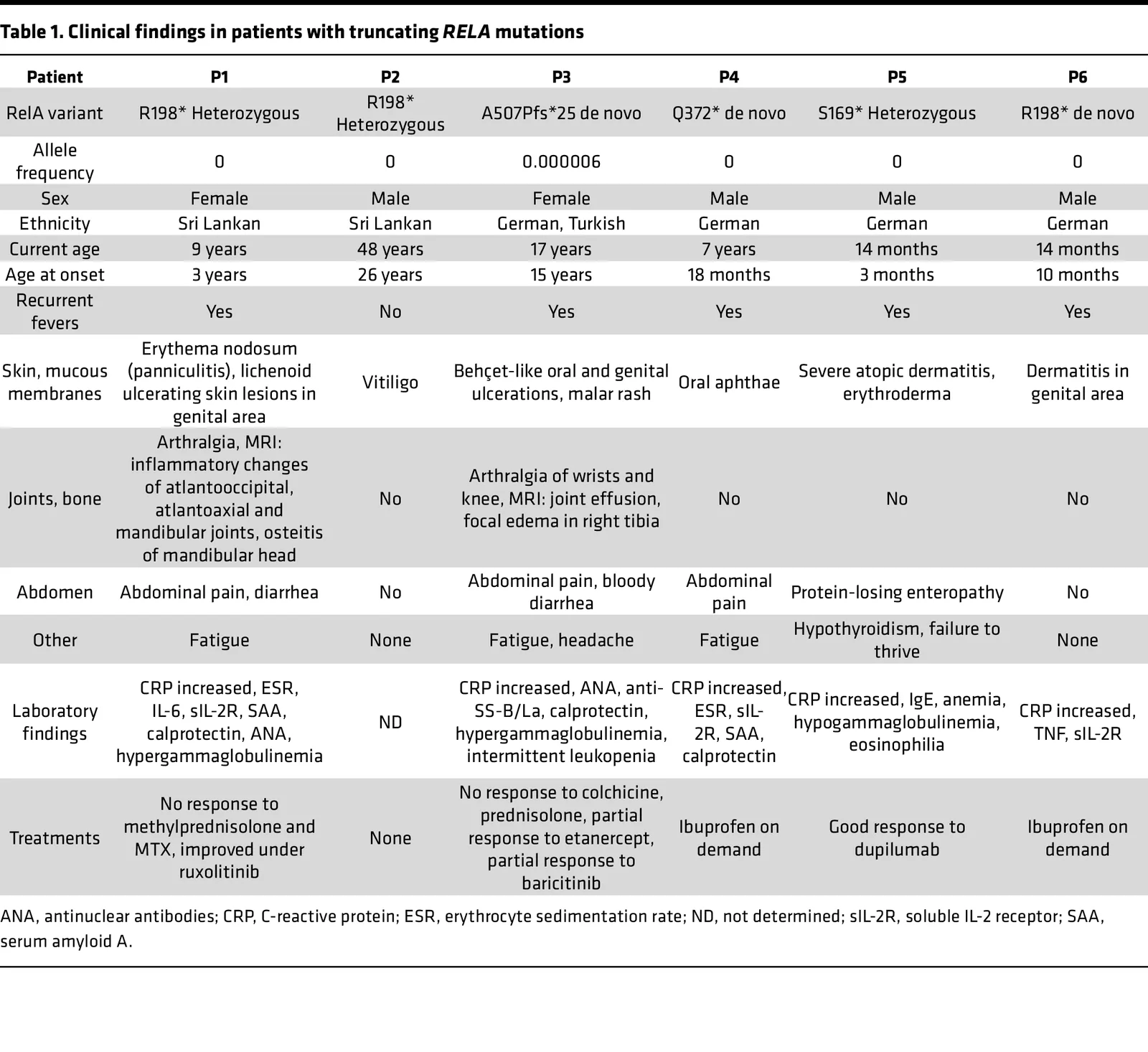
Clinical findings in patients with truncating *RELA* mutations

## Version 1 08/11/2026

In-Press Preview

## Version 2 09/22/2026

Electronic publication

## Version 3 09/25/2026

Correction to an author's name
