# Supplementary material for: Truncating RELA variants drive autoinflammation and autoimmunity by impairing the negative feedback control of NF-κB
Source: JCI Insight. 2026 Aug 11;11(18):e200002. doi: 10.1172/jci.insight.200002 (PMC13596719; doi:10.1172/jci.insight.200002)
Supplement: Supplementary file 1 [file jciinsight-11-200002-s011.pdf]

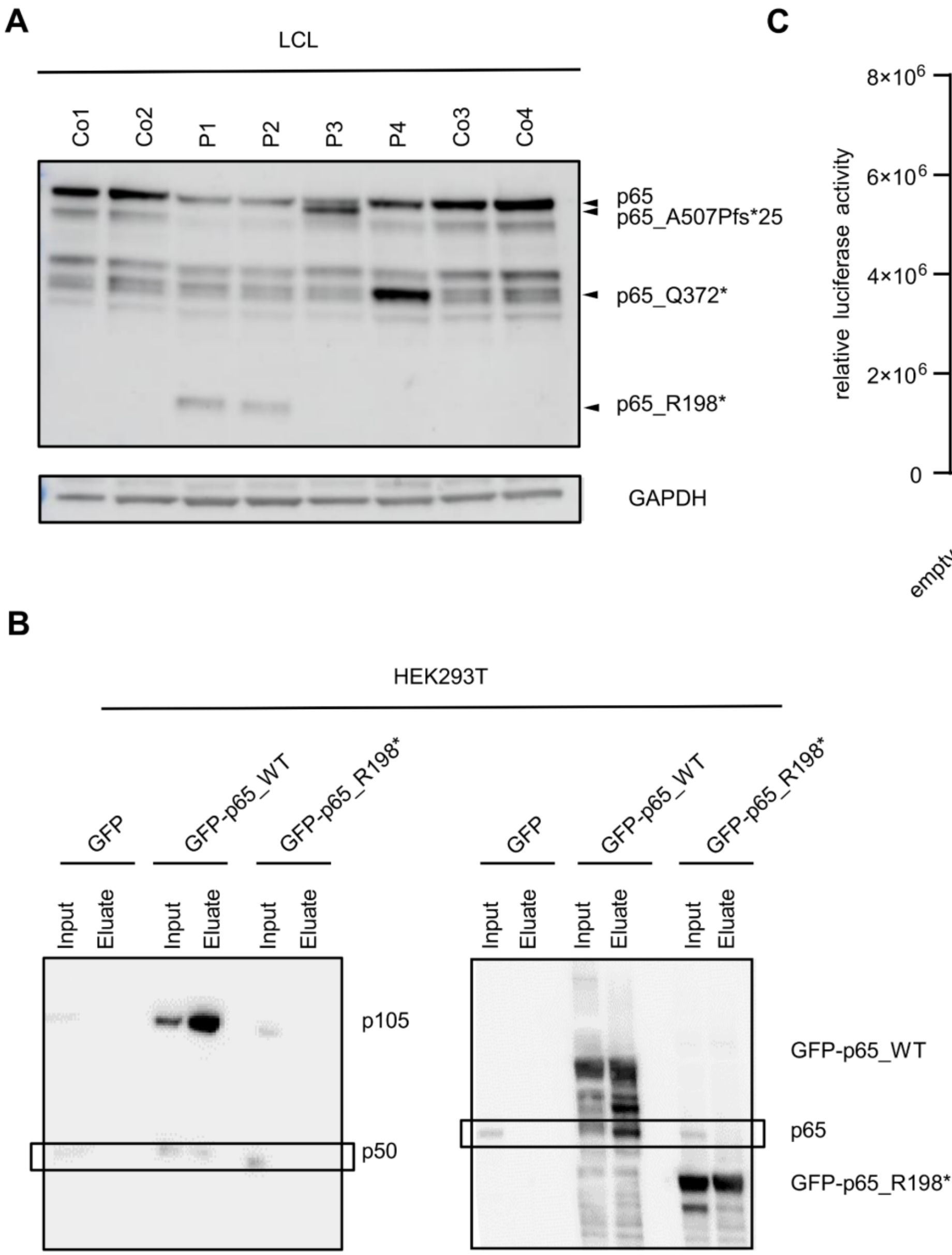

Supplemental Figure 1. Functional characterization of truncating p65 variants. **(A)** Expression of wild type (p65) and mutant p65 (p65_R198*, p65_Q372* and p65_A507Pfs*25) in patient LCLs (P1-P4) in comparison to four wild type controls (Co1-Co4). GAPDH was probed as loading control. **(B)** p65 homodimer and heterodimer formation assayed by co-immunoprecipitation of whole-cell lysates from HEK293T cells transfected with either GFP-tagged wild type (GFP-p65_WT) or mutant (GFP-p65_R198*) p65 using anti p65 antibody, followed by Western blot analysis using anti-p65 (homodimer) or anti-p50 antibody (heterodimer). **(C)** NF-κB activity measured by luciferase reporter assay in HEK293 TN cells expressing wild type (WT) or mutants (R198*, S169*, A507Pfs*25, Q372*) p65 or empty vector. Means ± SD. ****P < 0.0001 versus mean of wild type controls, two-way ANOVA (Dunnett's multiple comparison test). The experiment was repeated independently at least twice.

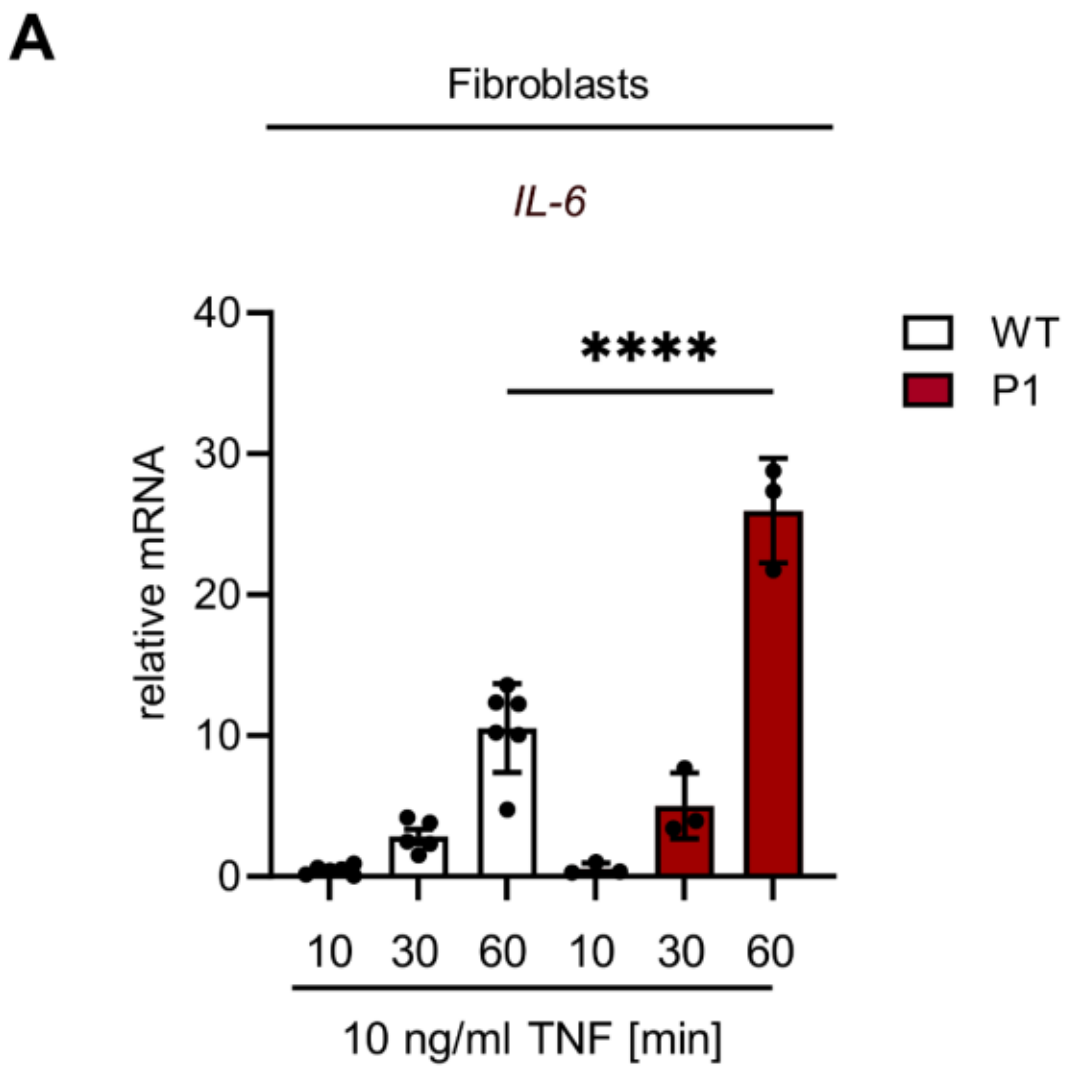

Supplemental Figure 2. **Patient fibroblasts are hyperresponsive to TNF stimulation. (A)** Relative expression of IL-6 in primary fibroblasts of patient P1 compared to two healthy controls (WT). Cells were stimulated with 10 ng/ml TNF for the indicated time periods. Data represent mean ± SD of three independent experiments. ****P < 0.0001 versus mean of wild type controls, one-way ANOVA (Šidák´s multiple comparison test).

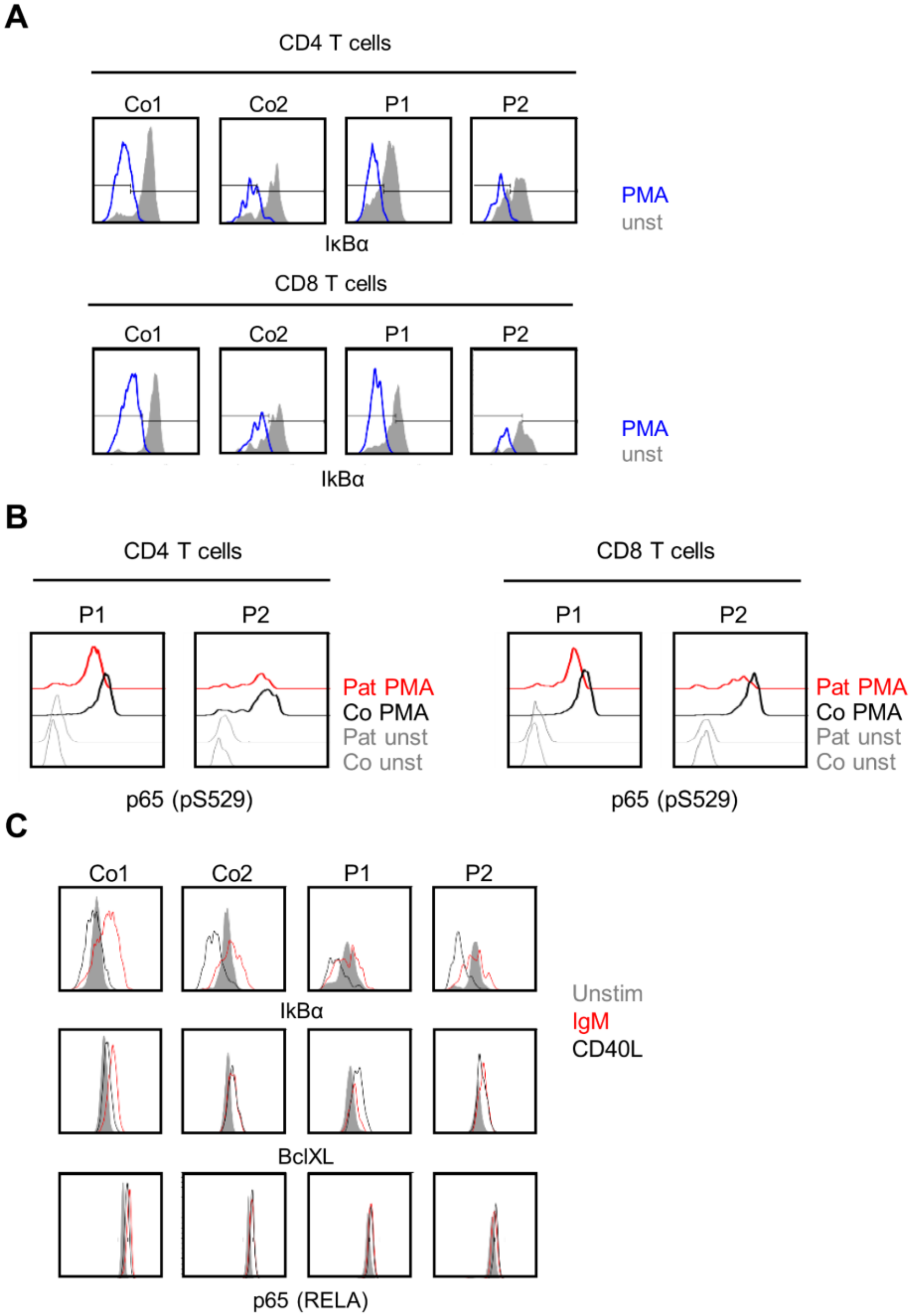

Supplemental Figure 3. Altered NF-kB signaling in patient PBMCs. (A) IκBα degradation in PMA stimulated CD4+ and CD8+ T cells from patients (P1, P2) compared to healthy controls (Co1, Co2). **(B)** Reduced p65 phosphorylation (pS529) in patient T cells upon activation of canonical NF-kB signaling. Mean fluorescence intensity (MFI) of phospho-p65 upon activation with PMA in CD4+ and CD8+ T cells of patients compared to healthy controls. **(C)** Protein expression of p65 and its targets IκBα (*NFKBIA*) and BclXL (*BCL2L1*) in PBMCs of patients and controls after stimulation with anti-IgM and CD40L for 36 h.

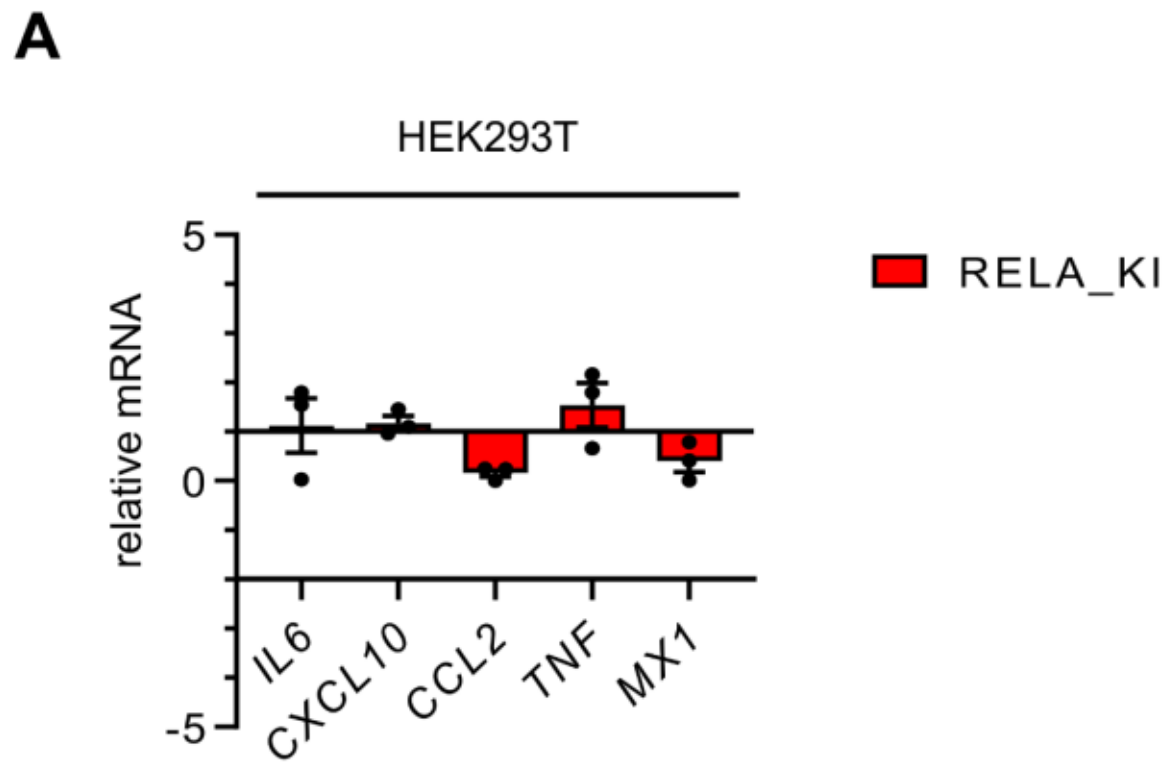

Supplemental Figure 4. Basal cytokine gene expression in heterozygous RELA^{R198*} knock-in HEK293T cells. **(A)** Relative expression of IL6, CXCL10, CCL2, TNF, and MX1 in unstimulated RELA^{R198*} knock-in HEK293T cells (RELA_KI) in comparison to wild type HEK293T cells, measured by qRT-PCR. Data are plotted as mean ± SD of three independent experiments.

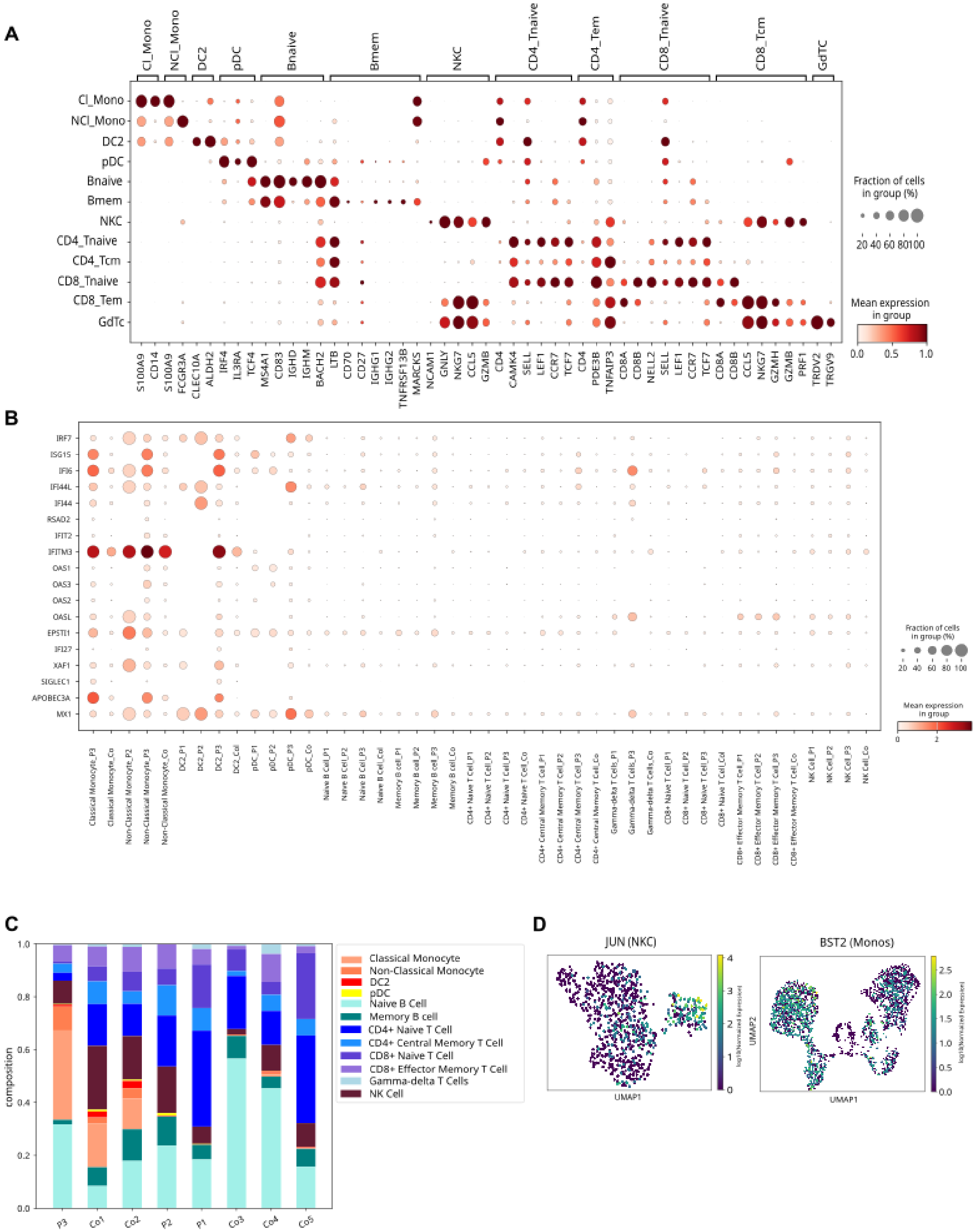

Supplemental Figure 5. Single-cell RNA seq analysis of PBMCs. (A) Dotplot showing the expression of marker genes used to annotate the indicated cell types. **(B)** Dotplot showing the expression of Type I interferon signature genes in patient and control cells of the indicated cell types. For controls, averages across all controls are displayed. **(C)** Barplot showing the cell type composition of patient and control samples. For patient 1 and 2 and controls (sc Co3, sc Co4 and sc Co5) monocytes were removed during FACS. **(D)** UMAP embedding of monocytes and NK cells, respectively, colored by the expression of the indicated genes in individual cells.

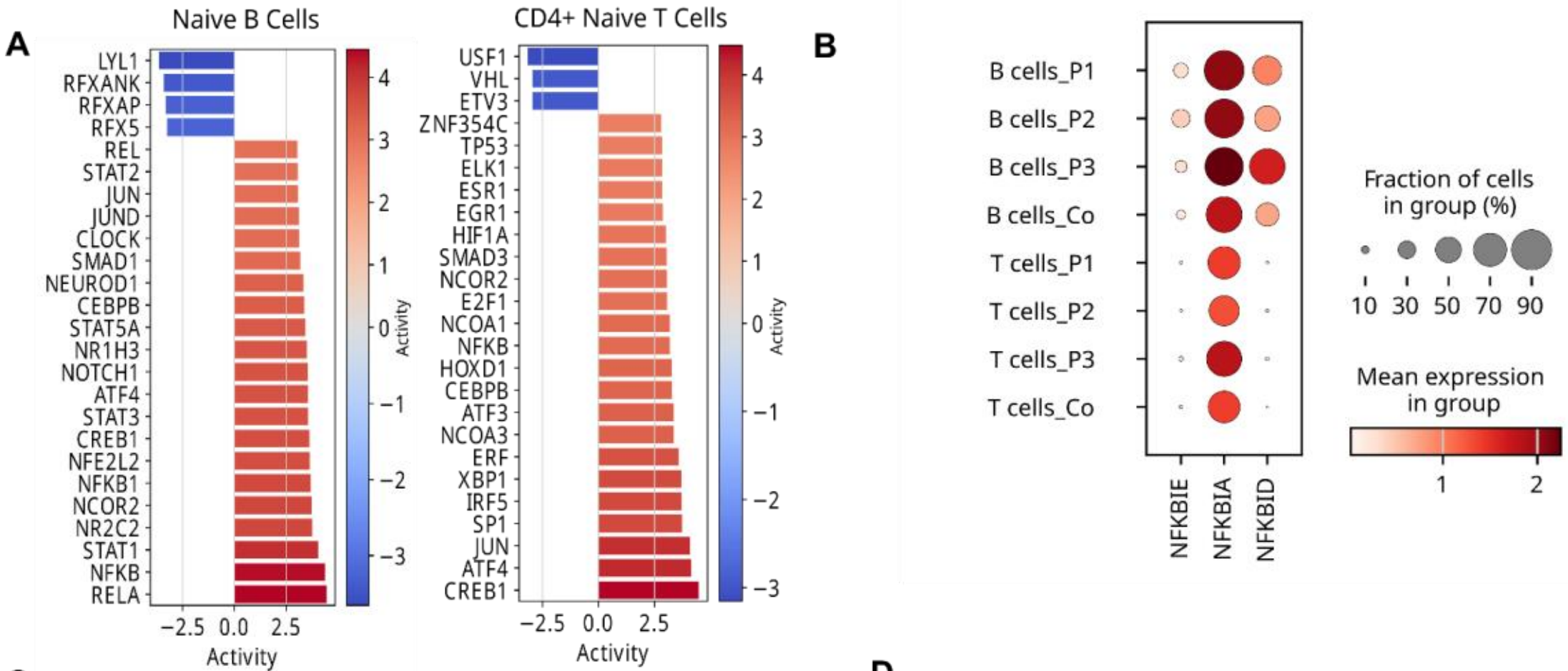

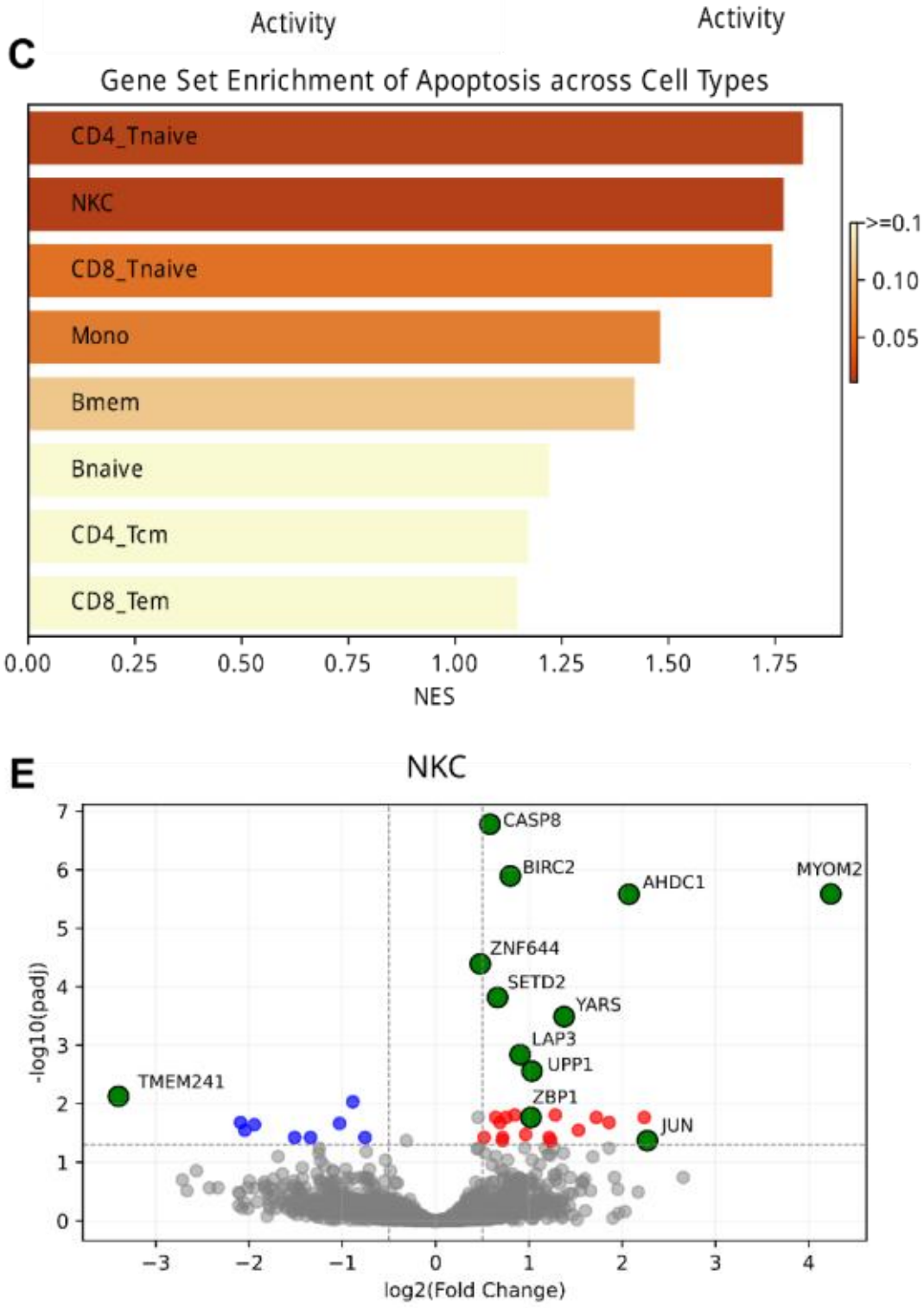

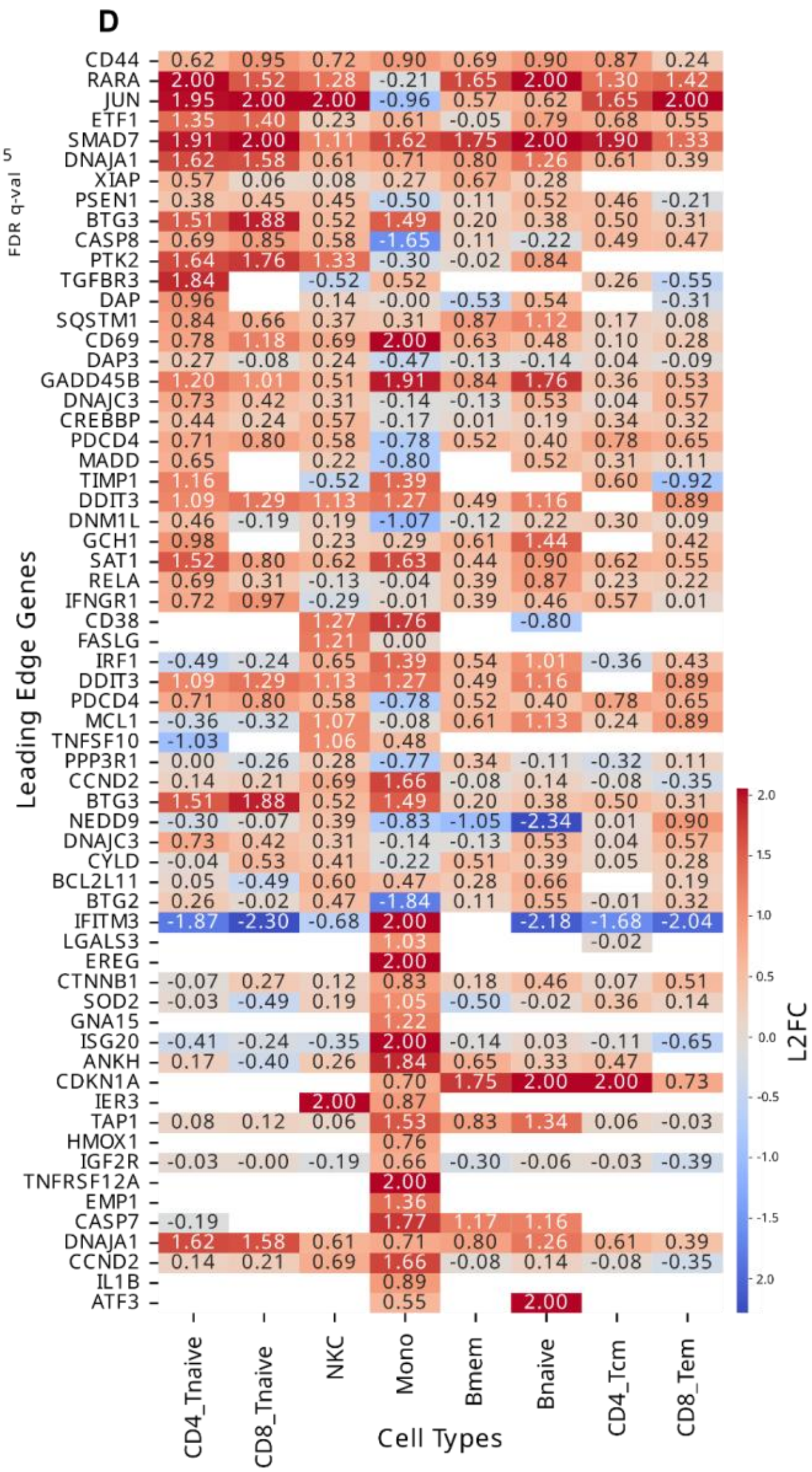

Supplemental Figure 6. Differential gene expression analysis of single-cell RNA seq data. (A) Bar plots reflecting relative transcription factor activity scores based on the expression of known targets of the indicated transcription factors for Naïve B cells and naïve CD4+ T cells respectively. **(B)** Dotplot showing the expression of NF-κB inhibitors in patient and control B and T cells. For controls, averages across all controls are displayed. **(C)** Barplot showing the Gene Set Enrichment Analysis (GSEA) enrichment of the MsigDB Term "Apoptosis" in differentially expressed genes between patient and control cells of the indicated cell types. FDR: False Discovery Rate, NES: Normalized Enrichment Score. **(D)** Heatmap showing the log2 fold-change in expression between patient and control cells for the GSEA "Apoptosis" leading edge genes derived from the analysis in T and NK cells as well as monocytes. **(E)** Volcano plot for the differential expression analysis between patient and control NK cells. The top 10 differential and biologically relevant genes are highlighted.

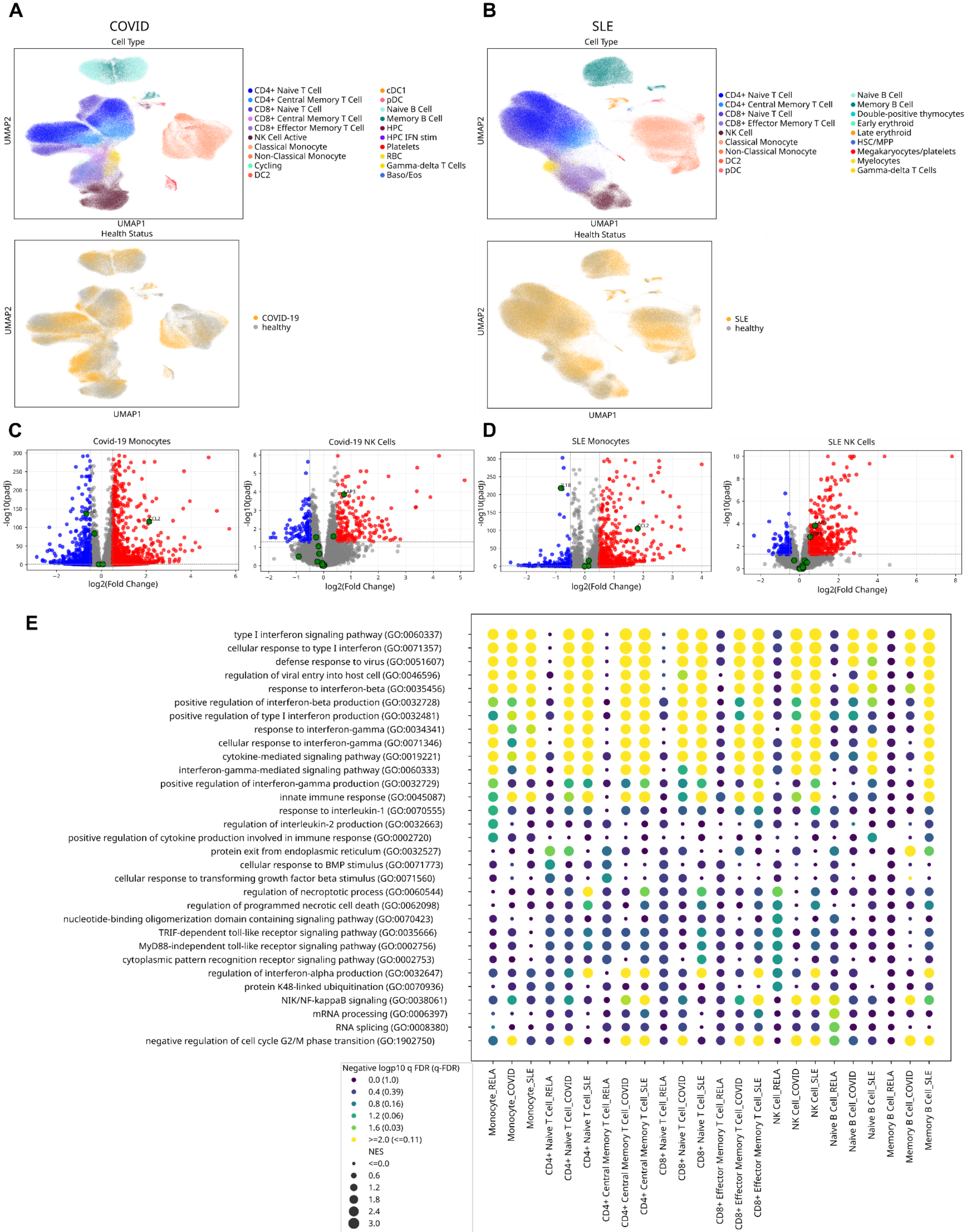

Supplemental Figure 7

Supplemental Figure 7. Comparative single-cell analysis of publicly available SLE and COVID-19 PBMCs. (A) UMAP embedding of scRNA-seq-profiled COVID-19 PBMCs, colored by cell type and health status. **(B)** UMAP embedding of scRNA-seq-profiled SLE PBMCs, colored by cell type and health status. **(C)** Volcano plots depicting differential gene expression between diseased and control COVID-19 monocytes and NK cells. Genes in green are upregulated in RELA mutation data. **(D)** Volcano plots depicting differential gene expression between diseased and control SLE monocytes and NK cells. Genes in green are upregulated in RELA mutation data. **(E)** Gene Set Enrichment Analysis (GSEA) of selected Gene Ontology (GO) Biological Processes terms in differentially expressed genes between patient and control cells for RELA, COVID-19, and SLE. FDR, False Discovery Rate; NES, Normalized Enrichment Score.
