# Supplementary material for: Truncating RELA variants drive autoinflammation and autoimmunity by impairing the negative feedback control of NF-κB
Source: JCI Insight. 2026 Aug 11;11(18):e200002. doi: 10.1172/jci.insight.200002 (PMC13596719; doi:10.1172/jci.insight.200002)

Full unedited gel for Figure 4C

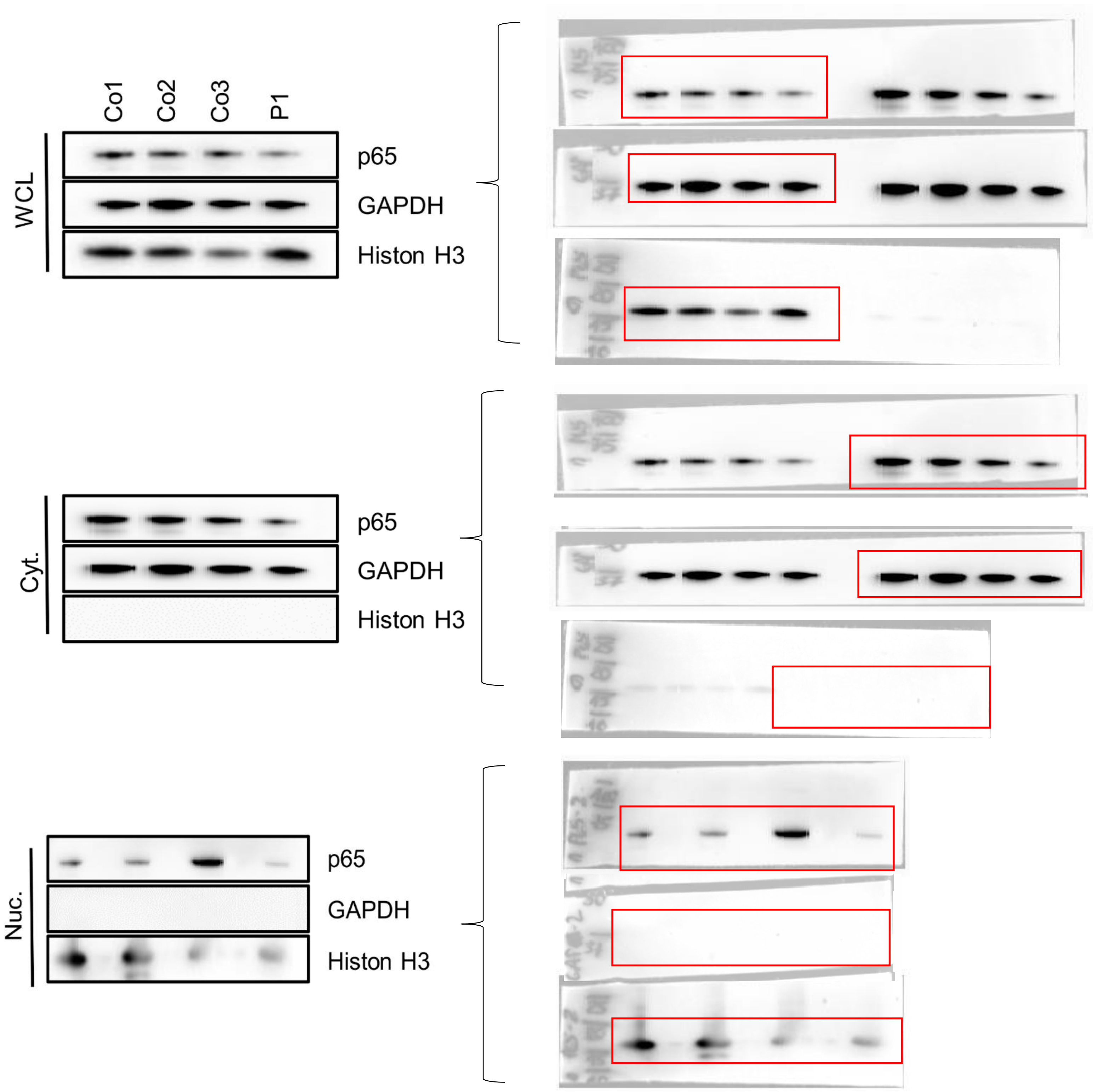

Full unedited gel for Figure 6A

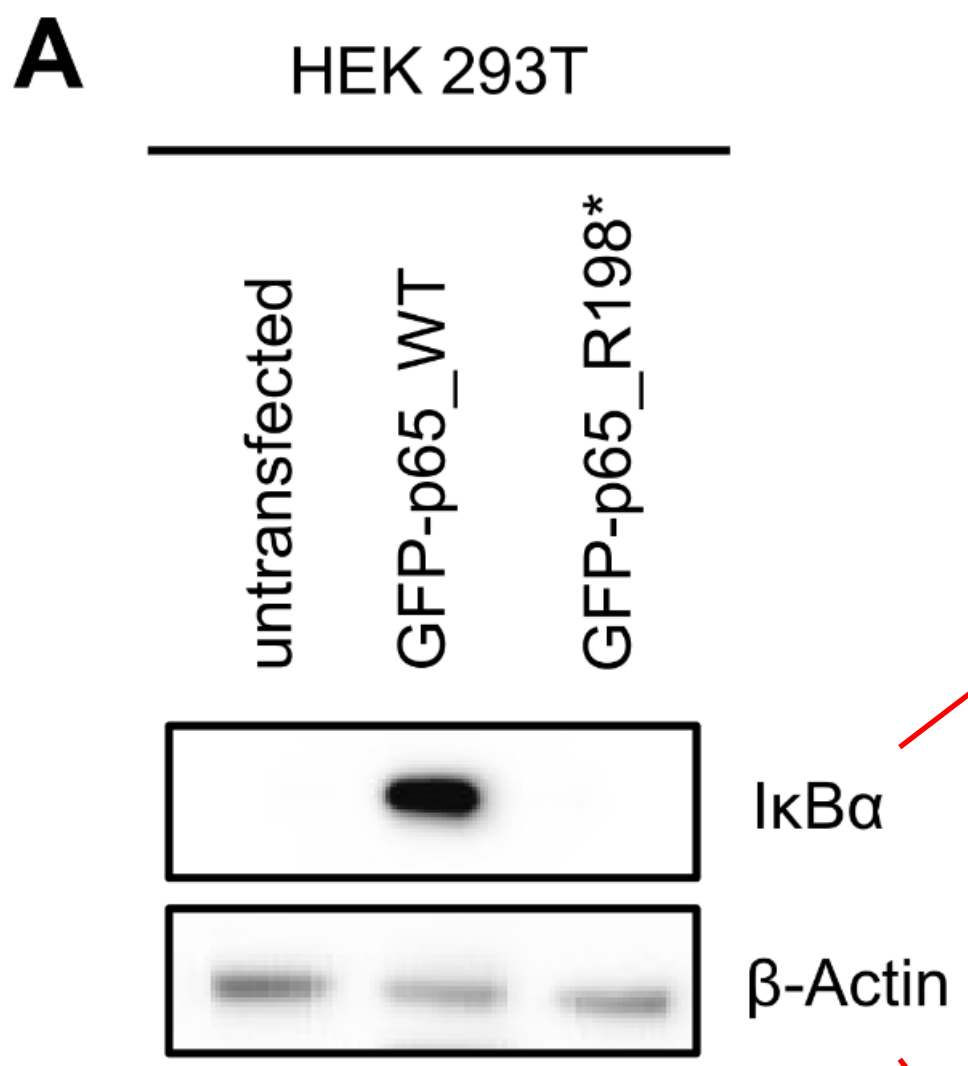

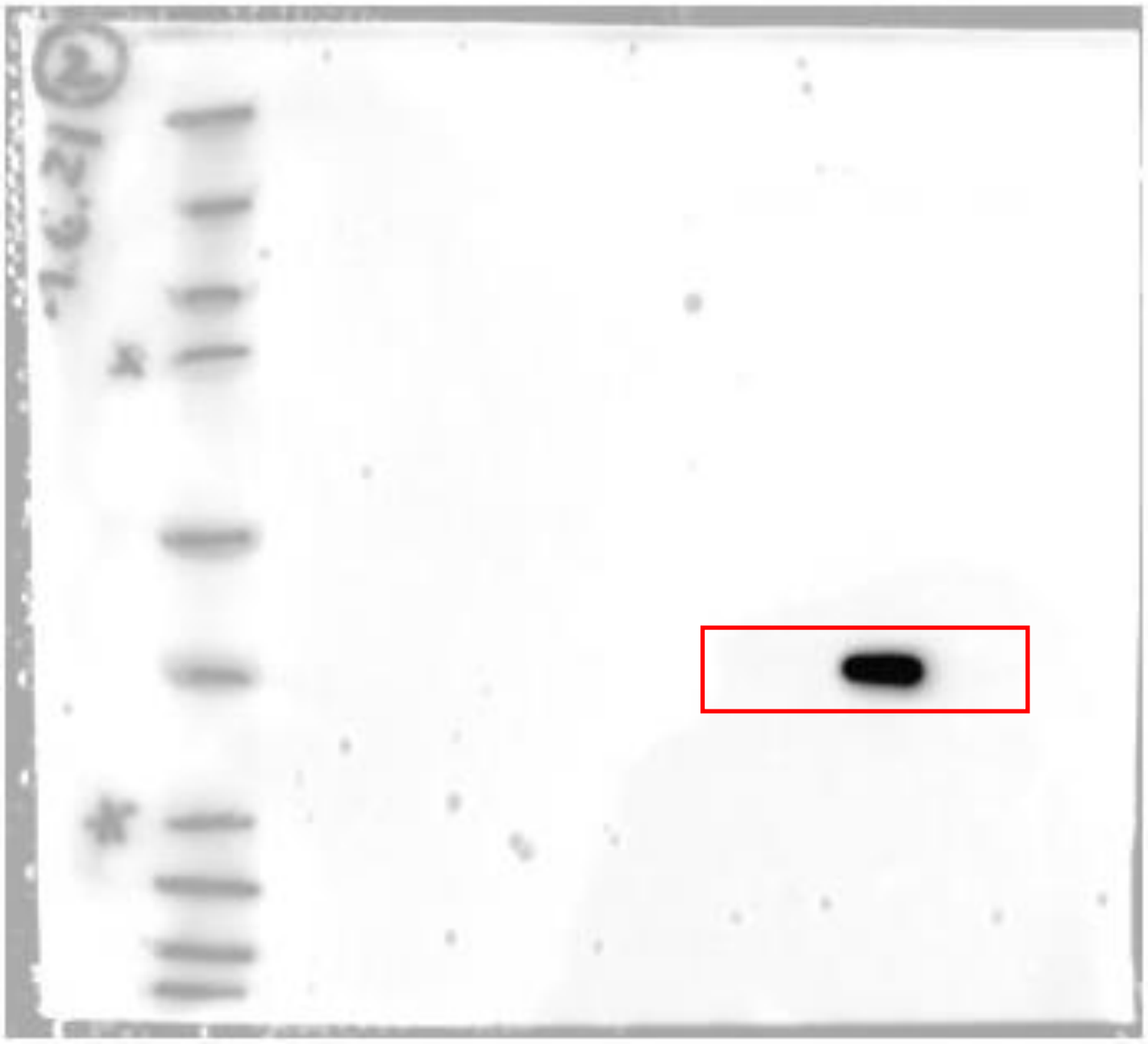

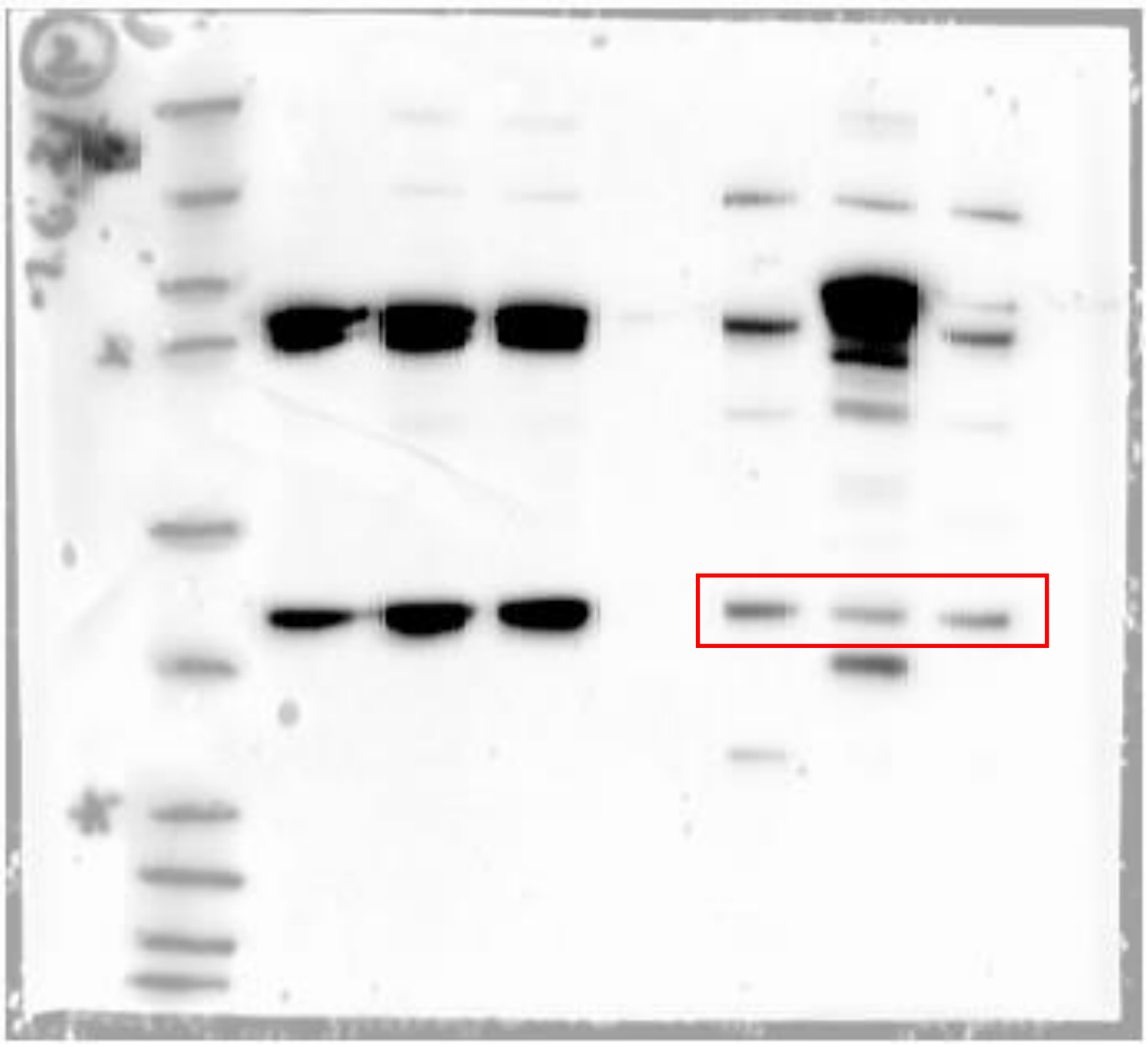

Full unedited gel for Figure 6C

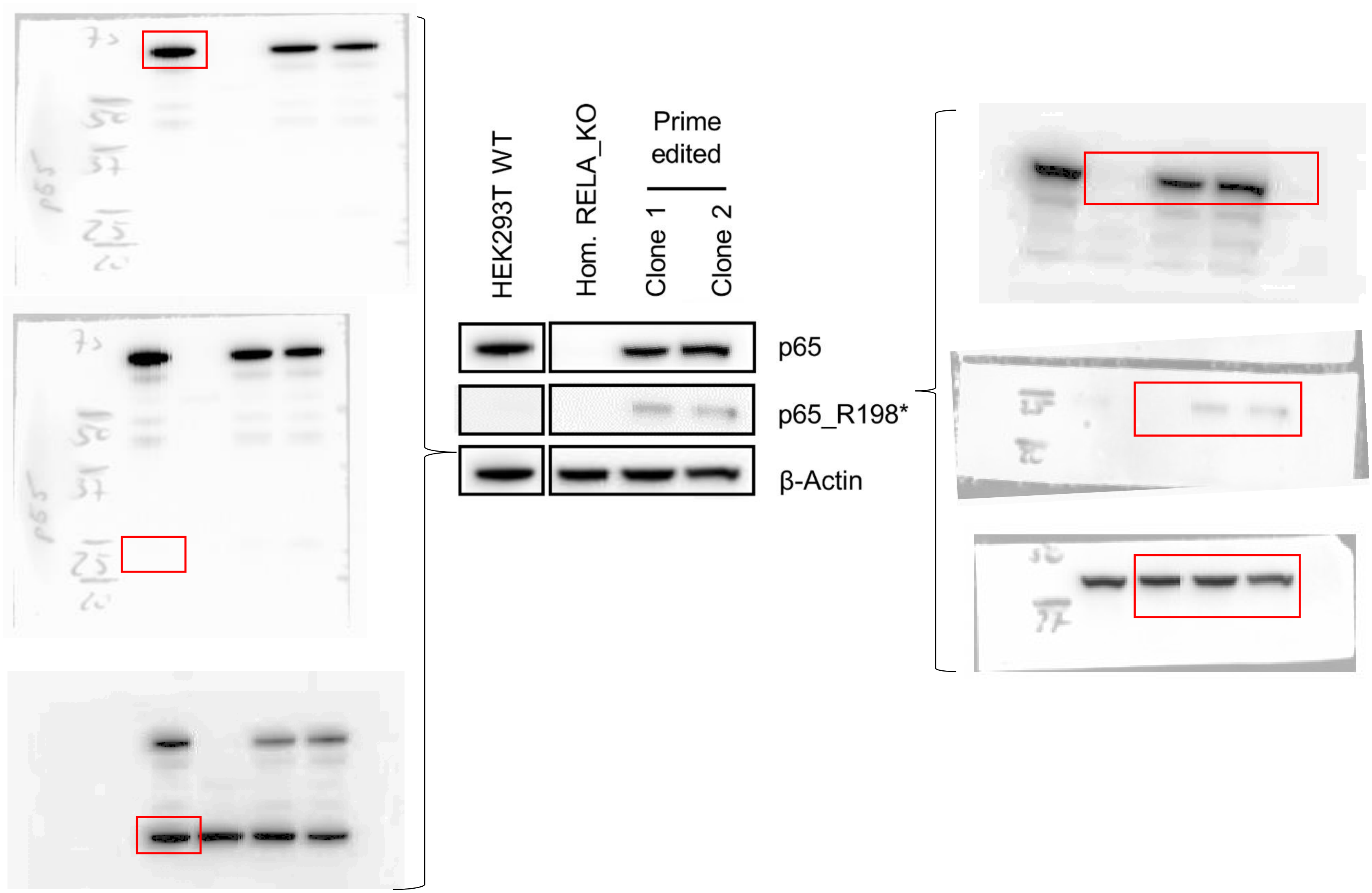

Full unedited gel for Figure 9A

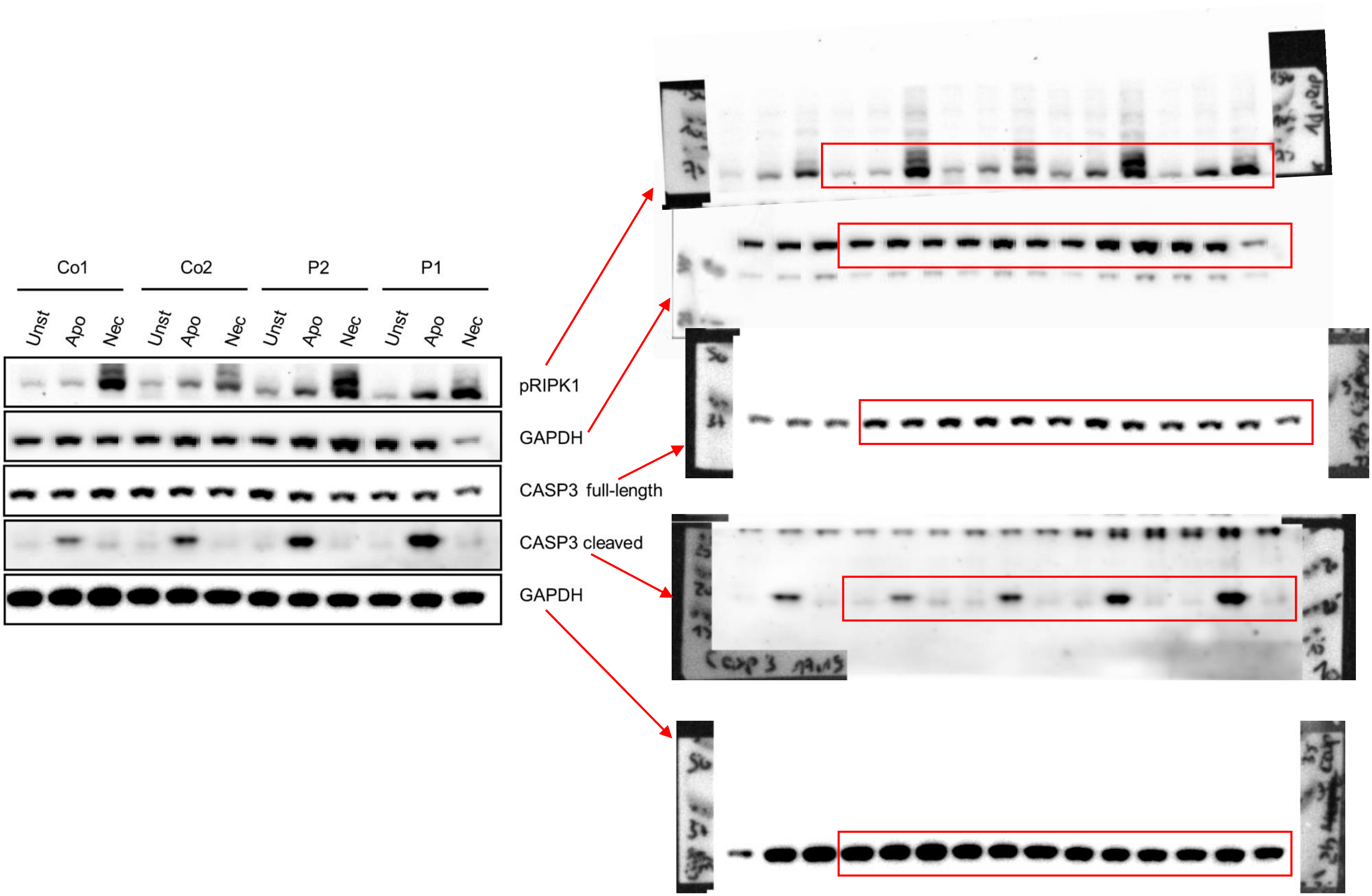

Full unedited gel for Figure 9B

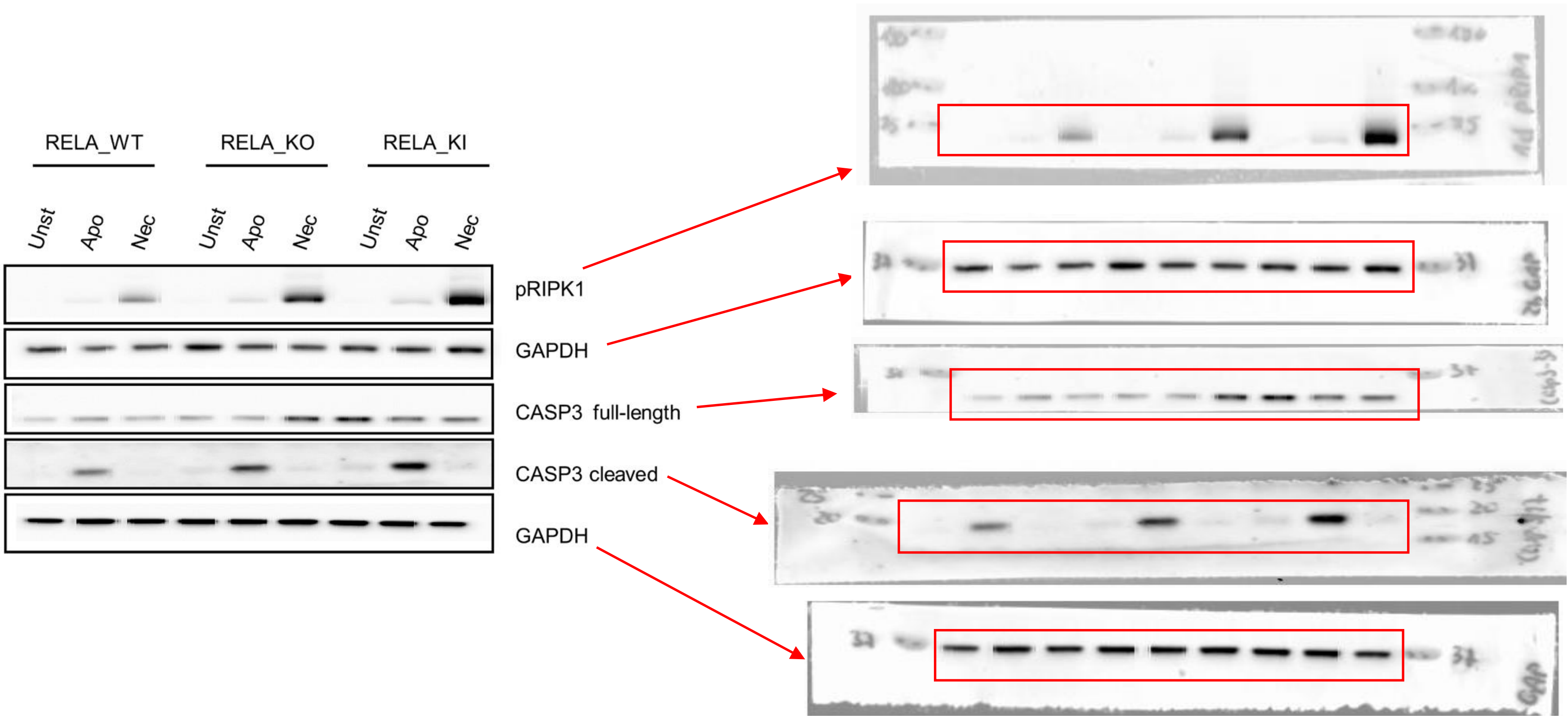

Full unedited gel for Supplemental Figure 1A

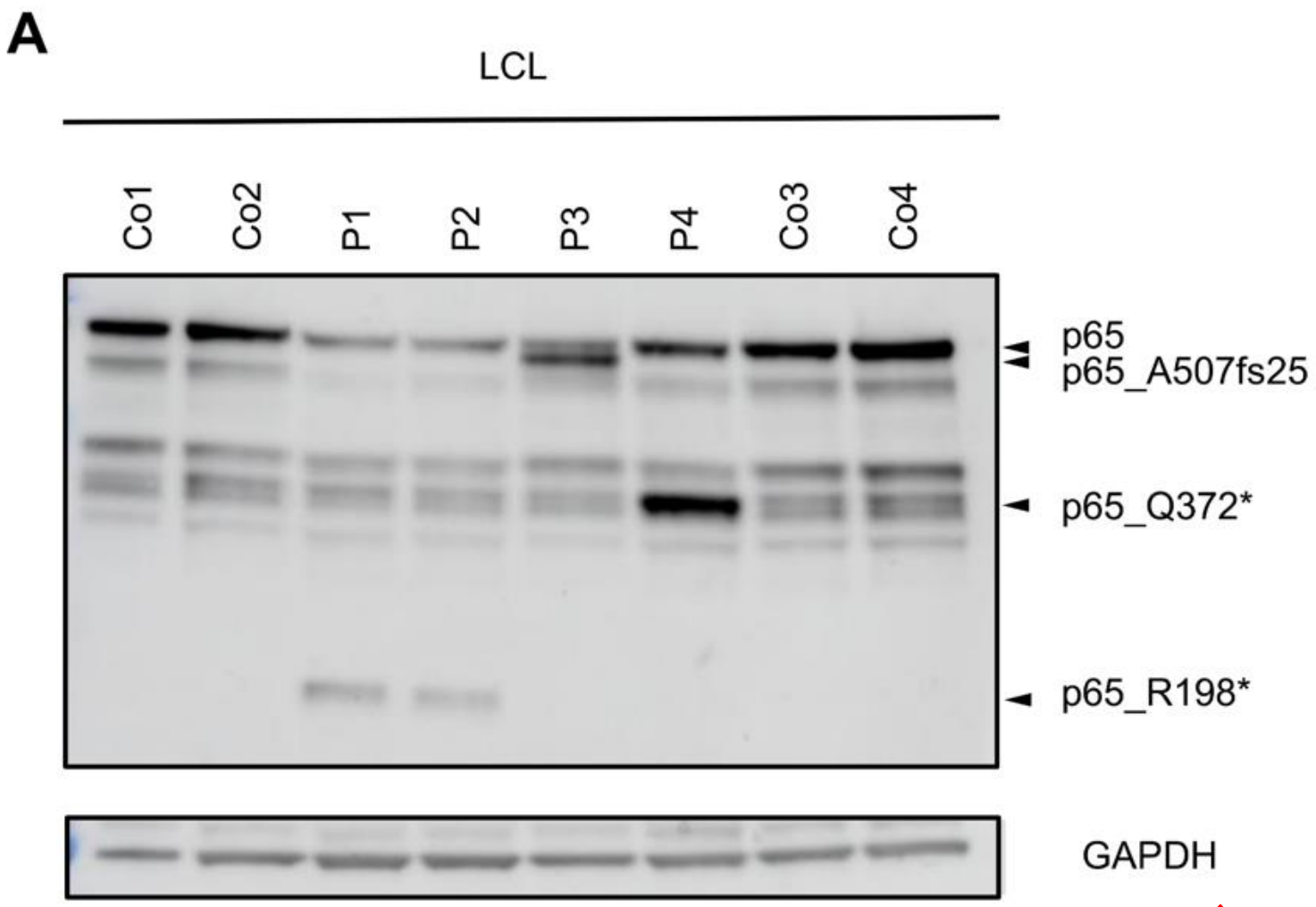

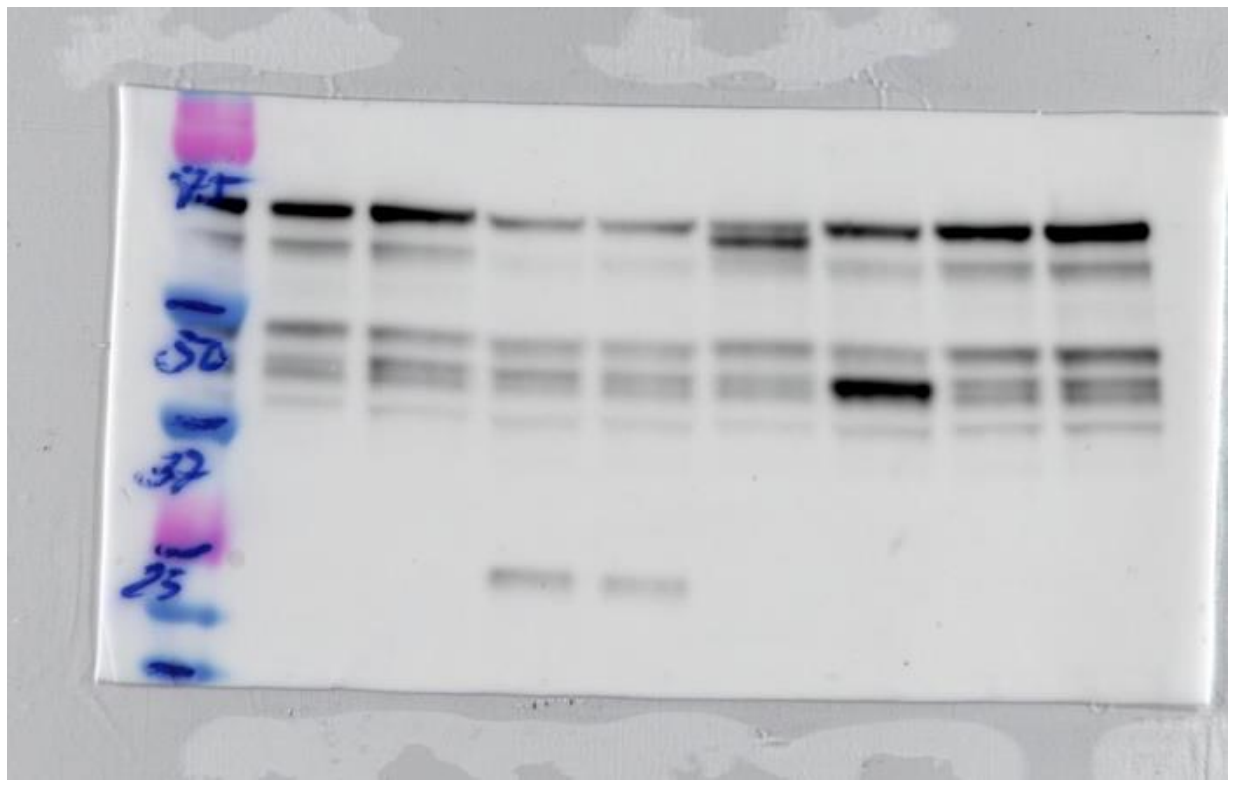

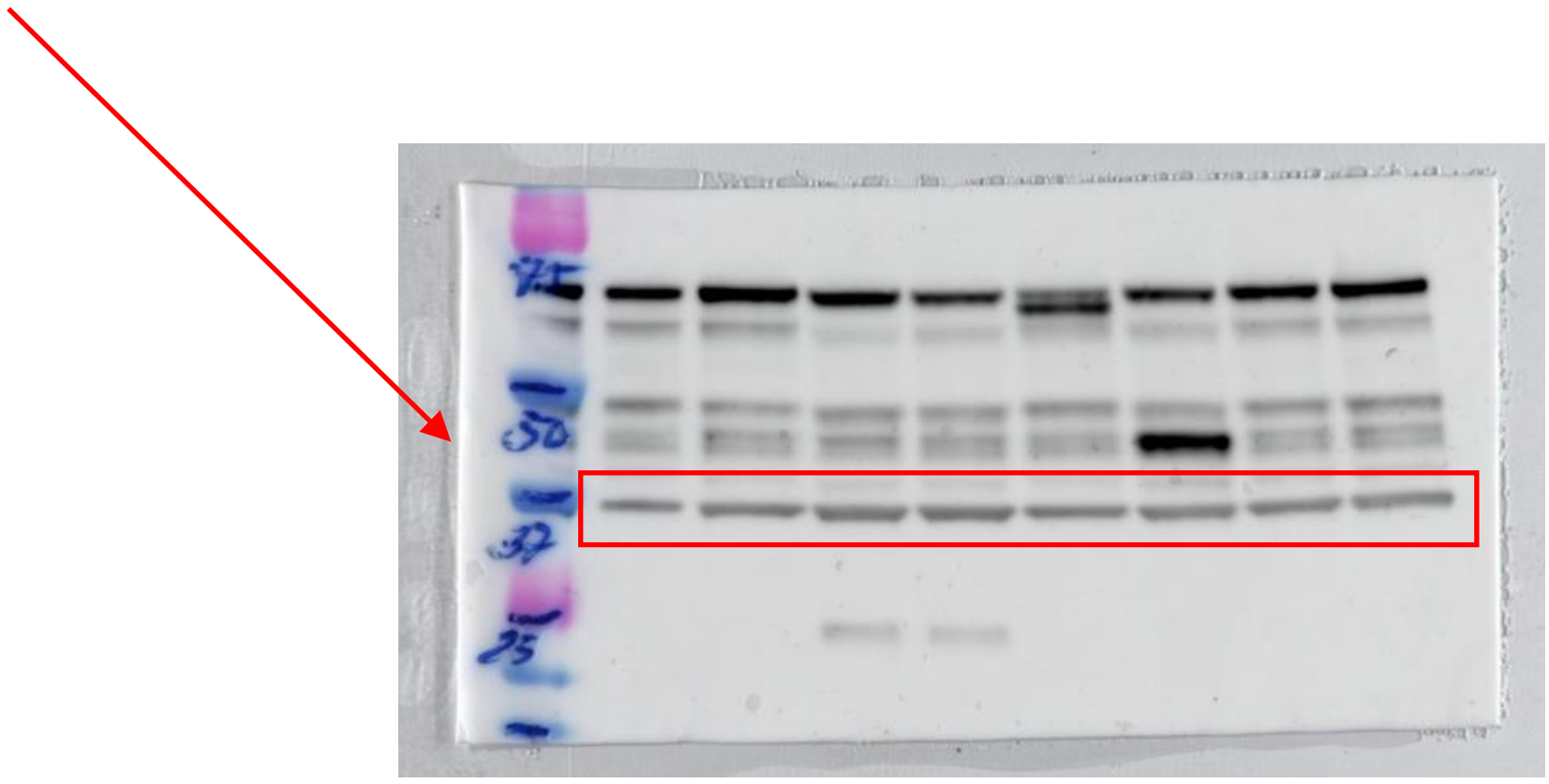

Full unedited gel for Supplemental Figure 1B

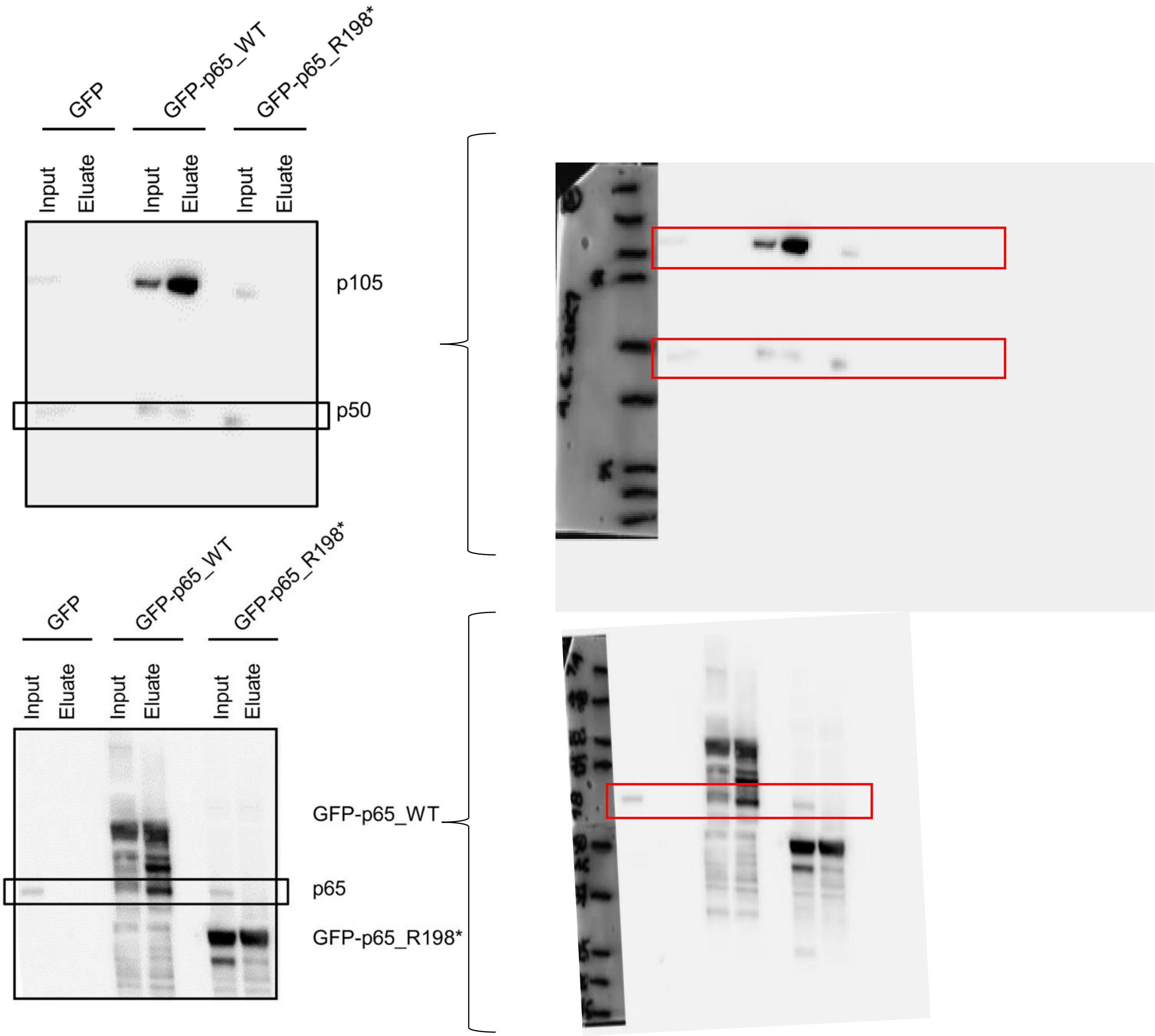

Supplement: Supplementary file 2 [file jciinsight-11-200002-s012.pdf]
